# Slow Harm After Dobbs v. Jackson Women’s Health Organization: An Analytic Scoping Review of How Data and Surveillance Infrastructures Reshape Emergency Obstetric Care

**DOI:** 10.7759/cureus.108911

**Published:** 2026-05-15

**Authors:** Angeline Ajit, Vanita Reddy

**Affiliations:** 1 College of Medicine, Texas A&M University, College Station, USA; 2 College of Arts and Sciences, Texas A&M University, College Station, USA

**Keywords:** abortion criminalization, dobbs, documentation practices, electronic health records, emergency obstetric care, health data interoperability, health information exchange, reproductive health privacy, scoping review, surveillance

## Abstract

Following *Dobbs v. Jackson Women’s Health Organization* on June 24, 2022, abortion criminalization in the United States has converged with interoperable health data systems, making reproductive health documentation more portable and legally legible across jurisdictions. This analytic scoping review, guided by Systematic Reviews and Meta-Analyses Extension for Scoping Reviews (PRISMA-ScR), examined U.S. literature published from June 2022 through October 2025 in PubMed, OpenAlex, Google Scholar, and HeinOnline. Sources were organized by evidentiary role into Tier C (legal and analytic conditions), Tier B (organizational adaptations), and Tier A (clinical impacts). The review mapped how surveillance pathways, including electronic health record and health information exchange interoperability, cloud hosting, billing and claims systems, and non-Health Insurance Portability and Accountability Act (HIPAA) consumer and law enforcement data, expand perceived liability and drive documentation and communication chill. Reported responses included vague documentation, reduced data capture, segmented records, and restricted information exchange. These adaptations may fragment care and delay intervention for emergency obstetric conditions. Tier A evidence included delays and higher diagnostic thresholds in ectopic pregnancy management, as well as increased maternal morbidity under mandated expectant management of previable preterm prelabor rupture of membranes and related second-trimester complications. These harms disproportionately affect patients experiencing structural racism, poverty, and intensified surveillance. We argue that these pathways constitute a form of slow harm, in which cumulative injury is produced through delay, fear, and institutional paralysis. Privacy-protective, equity-centered data governance and clearer clinical-legal guidance are needed to preserve safe documentation and coordinated emergency obstetric care.

## Introduction and background

The June 24, 2022, United States Supreme Court ruling in *Dobbs v. Jackson Women’s Health Organization* ended federal constitutional protection for abortion and shifted regulatory authority to individual states, leading to rapidly proliferating bans with wide variability in access and unclear exceptions [1]. Healthcare providers now face an unprecedented conflict: providing evidence-based obstetric care while navigating the threat of criminal and civil liability for themselves and their patients [2].

Critically, this legal regression in reproductive healthcare has unfolded alongside major advances in health data interoperability. Electronic health records (EHRs), health information exchanges (HIEs), and cloud-based storage systems have expanded the reach and portability of reproductive-health documentation [3]. Documentation is no longer confined to a single institution; information recorded in one setting may be accessed, interpreted, or subpoenaed in another [4].

Federal policies, including the HITECH Act and the 21st Century Cures Act, were designed to incentivize and, at times, require seamless data sharing. The United States Core Data for Interoperability (USCDI) formally defines pregnancy status as a core data element, reflecting its clinical importance, but without anticipating that aspects of pregnancy and abortion care could become criminalized [5]. This expanding interoperability exposes pregnancy-related information to legal scrutiny across jurisdictions, particularly in states with severe restrictions [3].

This convergence creates a structural tension, referred to here as the “Dobbs Double Bind,” in which protecting patient privacy conflicts with maintaining coordinated, high-quality care [6]. Clinicians may restrict documentation or communication to reduce perceived legal exposure, yet these same actions can fragment care and delay intervention in time-sensitive obstetric conditions. 

This review is guided by the concept of “slow harm,” defined as the cumulative injury produced through delayed care, fragmented communication, and constrained clinical decision-making under conditions of legal and institutional pressure. Rather than focusing on discrete adverse events, this framework captures how systemic delays and defensive adaptations can progressively degrade patient outcomes over time.

To examine how these policy and data dynamics operate in practice, we conducted a Preferred Reporting Items for Systematic Reviews and Meta-Analyses Extension for Scoping Reviews (PRISMA-ScR)-guided scoping review of U.S. post-*Dobbs* literature spanning legal analysis, organizational policy, and clinical outcomes. Using a tiered framework, we synthesized evidence across (1) legal and analytic conditions, (2) organizational and institutional adaptations, and (3) operational clinical impacts, with particular attention to emergency obstetric (E-OB) care.

Importantly, these burdens are not evenly distributed. Communities already subject to heightened surveillance, structural racism, and limited access to healthcare resources often have less capacity to absorb delays or mitigate data exposure, potentially exacerbating existing inequities in maternal health outcomes [7].

Our aim is not to estimate causal effects, but to synthesize mechanisms through which abortion criminalization and data governance reshape clinical practice. Synthesizing across tiers allows for a systems-level account of how punitive law and surveillance infrastructures re-engineer clinical documentation, information flows, and time-to-intervention in emergency obstetric care.​​ The guiding research question for this review is: *How do post-Dobbs surveillance and data governance structures produce mechanisms of slow harm in emergency obstetric care?*

## Review

Conceptual framework

Building on the definition introduced above, “slow harm” captures the temporal dimension of injury in post-*Dobbs *obstetric care, in which harm arises not only from outright denial of care but from systematic delay, fragmentation, and erosion of clinical discretion, with disproportionate effects on marginalized populations [7].

Reproductive Governance and Securitization

Reproductive governance describes how law, enforcement, and institutional discipline produce and legitimize these harms. After Roe’s reversal, states expanded criminal and civil enforcement, advancing reproductive governance through securitization [8].

This regime reshapes clinical practice by making ordinary medical communication and documentation feel legally actionable. Abortion bans frequently include criminal penalties for physicians and, in many states, provisions that empower private actors to track and punish violations, as exemplified by Texas’s Senate Bill 8 (SB8) [9]. For clinicians, this produces constant professional hypervigilance, described by OB-GYNs as feeling like “there’s a politician in the room” during patient conversations [10]. Providers worry about becoming test cases, “what if I say the wrong thing, what if it’s recorded, what if I don’t use the correct verbiage?” [10].

The “politician in the room” follows patients across the care continuum. The legal landscape creates potential criminal or civil liability for mental health providers (MHPs) who "aid and abet," "facilitate," or "advise" regarding abortion [11]. Chilling effects are driven by fear of being charged as an accomplice to murder, losing licensure, or facing civil lawsuits under "vigilante" laws. Even psychotherapist-patient privilege offers limited protection: prosecutors may attempt to invoke the “crime-fraud exception” in states that criminalize abortion to compel disclosure of psychotherapy sessions, arguing that therapy was used to aid a crime or tort [11].

Legal Risk and Clinical Chill

This environment chills both patients and providers [12]. Patients, aware of potential criminalization, may withhold information critical to their care or avoid seeking care altogether [2]. Even when seeking a legal abortion under exceptions like rape or incest, many state laws require victims to report the incident to law enforcement, a requirement that effectively bars access for the estimated 60% of rapes and 80% of incest cases that are never reported [1].

The resultant harm is often slow and insidious. Physicians in restrictive states report delays in medically necessary care until patients face severe deterioration. [13] Hospital legal teams are described as setting intervention thresholds so high that patients must become “septic or start hemorrhaging” before treatment can proceed [13]. This approach replaces nuanced, evidence-based decision-making with highly regulated counseling scripts driven by legal risk [14].

Over time, legal confusion can produce a “legitimacy paradox,” in which anti-abortion policy and culture make physicians in restrictive states less willing to provide or counsel on services even if legally permissible. This fosters avoidance, service contraction, and skill attrition, further thickening the conditions for slow harm [15].

Reproductive Justice and Disparate Impact

Surveillance exposure and delayed care intersect with the reproductive justice framework, where abortion access is inseparable from the right to parent children in safe, healthy environments [1]. Restrictive policies exacerbate existing structural inequities, as laws criminalizing pregnant people have historically targeted marginalized groups, particularly women of color and those who are economically disadvantaged [16]. Under* Dobbs*, the threat of criminalization, layered onto weak data privacy protections for reproductive health, has a distinct chilling effect for communities already subject to disproportionate surveillance and punitive state intervention [17].

Post-Dobbs data/surveillance pipelines

Enforcement increasingly relies on a vast, porous ecosystem of digital data pipelines that extend far beyond the Protected Health Information (PHI) covered by HIPAA, rendering many aspects of a person's reproductive life susceptible to surveillance [17].

EHR and Interoperability Pipelines

A growing “surveillance surface” spans medical and consumer pipelines, making reproductive health information (RHI) accessible, aggregable, and legally actionable. EHRs are a prime target because they contain highly reliable data on patient conditions, preferences, and treatment [2]. EHR systems are comprehensive, longitudinal, and increasingly interoperable, allowing data from clinical notes to imaging, laboratory results, and procedural codes to be accessed remotely across institutional boundaries [4]. Care documented legally in one state can be viewed and scrutinized in another where it is illegal [2].

Risk pathways within the formal health system operate through several interconnected mechanisms. HIEs, while essential for care coordination, also furnish complete patient records to a broad range of stakeholders, including laboratories and pharmacies [5]. In addition, reliance on cloud infrastructure means that data is physically hosted on remote servers, some of which may reside in abortion-restrictive states, thereby exposing records to legal process in those jurisdictions [3]. Insurance billing and claims systems create durable documentation trails that are difficult to obscure, and attempts to avoid these pathways, such as paying out of pocket, may deepen inequities for patients with limited financial resources [3]. These concerns are compounded by inter-jurisdictional conflict, as the uncertain constitutional protection of information contained in EHRs makes medical records potentially central evidence in extraterritorial litigation and criminal investigations, increasing institutional pressure to manage documentation defensively [2].

Consumer Data and Law Enforcement Pipelines Outside HIPAA

The most expansive pipelines operate outside of HIPAA, enabling Reproductive Health Surveillance. Period-tracking applications (PTAs) and other FemTech tools are often not “covered entities” and are governed primarily through the Federal Trade Commission (FTC) and uneven enforcement, leaving privacy protections dependent on after-the-fact policing and industry self-regulation [18]. The FTC’s 2020 complaint against Flo Health for sharing user data shows how easily reproductive data can be commercialized and repurposed [18].

Despite warnings to delete PTAs post-*Dobbs*, use has increased across surveyed states (adjusted prevalence ratio 1.20), suggesting that convenience, cycle management, or early pregnancy recognition outweighs perceived privacy risks, or that those risks are underestimated [19]. Together, regulatory gaps and rising uptake expand the surveillance surface available to restrictionist actors.

Outside HIPAA, reproductive-health surveillance operates through a broad set of digital pipelines that make intimate information collectible, inferable, and actionable. Data ecosystems routinely capture and monetize reproductive indicators such as period-tracking application data, search history, GPS location, and online purchases [20]. Triangulation of these non-health data can infer pregnancy and outcomes (e.g., pregnancy test purchase followed by resumption of alcohol consumption) [1]. Law enforcement can deepen this exposure through geofence and keyword warrants, which enable reconstruction of users’ movements and online queries from digital breadcrumbs [21]. Crisis pregnancy centers (CPCs) represent an additional nontraditional pipeline because they are often not staffed by medical professionals, are generally not bound by HIPAA, and may compile “digital dossiers” later exploitable by restrictionist actors [22]. Emerging reproductive-health tools, including Large Language Models and chatbots, introduce further privacy, ethical, and safety risks through data logging, misinformation, and insecure management of sensitive disclosures [23]. At the same time, machine-learning inference techniques can infer pregnancy status from heterogeneous datasets without direct tracking, widening the scope of legally actionable information [17].

These technologies intensify slow harm by shifting surveillance upstream, “reveal[ing] the user’s thoughts before they act on them” [12].​​​ Search history thus creates an evidentiary trail long before a clinical diagnosis, and the resulting anticipatory fear helps set in motion the documentation chill and delayed intervention described in Mechanisms I and II [12].

Mechanisms I: documentation and clinical communication

Surveillance and criminalization have restructured how clinicians document care and communicate with patients, activating slow-harm pathways through documentation changes and counseling/disclosure chill.

Chilling of Clinical Communication

The threat of scrutiny undermines the trustful patient-physician relationships linked to improved outcomes [2]. OB-GYNs describe governmental intrusion into their relationship with patients and a loss of the ability to speak freely [10]. Among pediatric primary care providers (PCPs), 81% felt Emergency Contraception prescription was more important post-*Dobbs*, yet only 7% reported increasing prescribing, an operational disconnect consistent with systemic and knowledge barriers and a broader “chill” on care [24].

This chilling effect is not limited to clinicians. Patients may delay or avoid seeking care, which is the fourth leading contributing factor to pregnancy-related deaths [25]. They may also withhold sensitive but clinically relevant information, including substance use, smoking, or alcohol use, because they fear those disclosures could be used against them [26]. Some patients further report explicitly weighing the potential legal or professional consequences to their physicians when deciding whether to pursue abortion care [10].

Restructuring Documentation (Operational Evidence - Tier A/B)

In response to these risks, providers and institutions have adopted ad hoc and formal documentation changes, recognizing that EHR documentation is searchable and accessible [2].​​​ These responses generally fall into two overlapping patterns: minimization through vague language and organizational mitigation strategies designed to reduce PHI exposure.

With respect to minimization, genetic counselors have reported making their documentation more “vague” or “generic” and using “code words” agreed upon by colleagues to render clinical notes inconspicuous [27]. Some have also avoided documenting conversations about abortion in the patient chart altogether, relying on phone calls instead [28]. Post-SB8 patient narratives similarly describe a breakdown in the therapeutic patient-physician relationship, including an implicit “gag rule” in which termination becomes unspeakable within clinical settings, thereby shifting the burden onto patients to seek information independently and self-refer outside formal systems, a burden that falls most heavily on those with fewer resources [29]. Institutions have likewise advised clinicians to avoid documenting pregnancy status in the early first trimester or while abortion is under consideration [3]. When documentation is necessary, providers are urged to use nonspecific language and the most generic diagnosis and procedure codes possible to balance clinical specificity against legal risk [3].

Comparable changes appear in other specialties. Nuclear medicine departments, for example, have been advised to revise blanket policies requiring routine collection of RHI and instead limit collection to select cases (e.g., I-131, therapeutic procedures) to mitigate legal risk [30]. Radiologists have also reported hedging or drafting ambiguous reports out of fear of scrutiny-ambiguity that can itself delay care when treating providers seek additional tests to resolve uncertainty [31]. Professional guidance has extended these practices into mental health and telehealth settings as well. For example, the APA has advised MHPs to document vaguely, using terms such as “family issues” or “healthcare decisions,” and to avoid wording that could be construed as abortion referral or assistance, while providers in the South report that fear of litigation under unclear telehealth laws has made telehealth abortion a lower institutional priority because it could place an “even larger target” on their clinics [32]. Legal uncertainty also reaches fertility care: laws concerning personhood (e.g., Alabama’s frozen-embryo decision) are prompting clinicians to modify how they discuss preimplantation genetic testing for aneuploidy and embryo disposition, demonstrating that uncertainty around embryo status can directly alter IVF decision-making and risk communication [33].

At the organizational level, institutions adopt policies to reduce PHI exposure. Some health systems classify pregnancy and abortion care as “highly protected,” restricting electronic exchange (e.g., with Care Everywhere) and requiring explicit consent for Release of Information (ROI) requests [3]. Others use segmented or parallel documentation, separate EHR instances or paper records, for abortion episodes, but this can disrupt continuity (e.g., missing allergies/medications) and may still be subpoenaed [3]. Institutions may also relocate cloud servers to protected states and offer out-of-pocket payment to bypass insurance-claim pipelines, though this risks exacerbating financial disparities [3]. Finally, Health Care Organizations (HCOs) are encouraged to enter DUAs (data use agreements) with Business Associates to require policy compliance and notification before releasing records sought by prosecutors [34].

Mechanisms II: time-to-intervention in E-OB

A clinically consequential result of defensive documentation and institutional fear is the mandatory delay in time-to-intervention for emergency obstetric (E-OB) conditions, transforming standard, time-sensitive treatments into life-threatening emergencies.

Ectopic Pregnancy Delays (Tier A)

Ectopic pregnancy is a nonviable condition that can become life-threatening without timely intervention, and the standard of care is prompt medical or surgical termination [35]. However, Post-*Dobbs* restrictions have compromised emergency management of this condition. A national survey of emergency physicians found that 24% of respondents in restrictive or semi-restrictive states reported delays in the management of known or suspected ectopic pregnancy since *Dobbs* [35]. In the same survey, 54% reported adapting care to remain within legal parameters, most often by requiring a higher degree of certainty before making a definitive diagnosis; 58% identified this heightened certainty threshold as the primary reason for delay [35]. Reported adaptations included additional imaging (26%) and serial β-hCG testing (24%) before treatment, sometimes using thresholds above American College of Obstetricians and Gynecologists (ACOG) guidance (e.g., β-hCG >4,000), thereby increasing the risk of discharging patients who need timely, definitive care [35]. Qualitative case narratives describe the resulting slow harm: patients delaying local care due to fear, then arriving out of state with ruptured ectopic pregnancies requiring major surgery, incurring high costs, and recovering away from their support systems [7]. The Supreme Court’s failure to rule decisively on the Idaho EMTALA case has further prolonged uncertainty over whether EMTALA preempts restrictive state bans [25].

Second Trimester Complications and Morbidity (Tier A)

When abortion laws provide only narrow exceptions for maternal life, clinicians may be compelled to wait until a patient’s condition escalates to emergency signals, including sepsis or hemorrhage, before intervening. A review of two Texas hospitals post-SB8 found that state-mandated expectant management of complications like Premature Prelabor Rupture of Membranes (PPROM) before 22 weeks was linked to substantial maternal morbidity: serious maternal morbidity occurred in 57% of expectant-management patients versus 33% among those who elected immediate termination in states without such restrictions [14]. Narrative evidence from the Care Post-Roe study reinforces these findings of slow harm. One patient with PPROM at 16 to 18 weeks was denied abortion, discharged, and later developed sepsis requiring ICU care and a five-day hospitalization [7]. Another patient with PPROM drove nearly four hours for self-transfer after five days of ruptured membranes and presented only after cord prolapse occurred, with the cross-state coordination burden also diverting resources from other patients [7]. In another case, a patient carrying twins developed HELLP syndrome (a life-threatening form of preeclampsia) after one twin died. Because termination could not be offered in the banning state, she was transferred, deteriorated during transport, lost the second twin, and the delay was documented as a “near-miss” [7]. A further case described a patient with severe postpartum cardiomyopathy who obtained a needed D&E (dilation and evacuation) only after a six-week delay for an out-of-state appointment, pushing the procedure into the mid-second trimester and heightening her risk of death [7].

Institutional and Training Fragmentation (Tier B/C Compounding Effects)

These delays are compounded by organizational failures and knowledge deficits. Providers report a lack of clear guidance and support from hospitals, leaving clinicians to navigate legal and clinical uncertainty alone [36]. Transfers between systems, driven by divergent institutional interpretations of ambiguous statutory language, disrupt continuity and increase delays and morbidity [7]. At the same time, emergency nurses report training deficits around E-OB care, exacerbating risk as emergency departments become default sites for unplanned OB care due to expanding maternity care deserts [37].

Disparate harms and who is most affected

The restructured care environment and resulting slow harm disproportionately burden marginalized populations, amplifying structural racism and socioeconomic inequities [25]. Patients facing higher rates of poverty have fewer resources to afford out-of-state care or telehealth services, and the logistical burdens of travel, lodging, childcare, and time away from work fall most heavily on low-income, Black, Brown, and Indigenous patients, at times making care inaccessible altogether [25]. Even digital privacy mitigation (VPNs, privacy-focused tools) is often expensive, making the ability to shield oneself from surveillance a privilege of the socioeconomically advantaged [17].

These inequities are layered onto a longer history in which the criminalization of pregnancy outcomes has disproportionately targeted low-income women of color [17]. For instance, in reproductive settings, Urine Drug Tests (UDTs) can operate as stigma-based surveillance, where a positive result is treated as grounds for withholding care and can trigger child welfare investigations and family separation [38]. Clinicians are documented to test and report Black pregnant people more than White pregnant people, increasing adverse outcomes for minority patients [38]. Surveillance exposure is also unevenly distributed by digital literacy, immigration status, and proximity to carceral systems, meaning the same documentation and pipeline risks can compound existing vulnerabilities even where direct empirical measurement is still limited.

These burdens are intensified by unequal baseline maternal risk and unequal margin for delay. Black women already experience markedly higher maternal morbidity and mortality, and states with restrictive abortion policies tend to have worse maternal and infant outcomes [6]. When time-to-intervention is structurally delayed, groups with less physiologic and social margin for delay bear disproportionate consequences. Provider narratives reflect this distribution: in Care Post-Roe narratives, patients described as Black or Latina/Latinx/Hispanic accounted for about half of cases documenting deviation from standard care due to abortion restrictions [7]. Adolescents, who account for a high percentage of abortion seekers, also face unique barriers related to travel, cost, and parental involvement laws [25].

For marginalized communities, the threat of legal action and surveillance erodes trust in providers and health systems, intensifying longstanding medical mistrust rooted in historical disparities [16]. As a result, individuals-especially those already subjected to surveillance and criminalization-may avoid routine care and broadly chill use of health-related technologies such as telehealth, further limiting access to preventive and acute care [26]. This systemic fear reinforces the two-tiered system of healthcare that existed even under Roe's promise [1].

Methods (scoping review)

This paper synthesizes evidence from a focused scoping review (PRISMA-ScR) of U.S. literature published from June 24, 2022 (*Dobbs* decision date) through October 7, 2025. Subsequent policy and legal developments are noted in the Discussion to contextualize the model but were not included in the systematic search, screening, or tier assignment. Searches spanned (1) medical databases (PubMed/MEDLINE), (2) academic indexing databases (OpenAlex), (3) multidisciplinary search engines (Google Scholar), and (4) legal/policy archives (HeinOnline).

Search terms combined three concept domains: (1) reproductive policy (“Dobbs,” “post-Roe,” “abortion ban”); (2) data/surveillance infrastructure (“EHR,” “HIPAA,” “health information exchange,” “privacy,” “law enforcement”); and (3) clinical or documentation outcomes (“documentation,” “counseling,” “time to intervention,” “PPROM,” “ectopic pregnancy,” “sepsis”).

A representative PubMed search string is provided below:

(“Dobbs”[tiab] OR “post-Roe”[tiab] OR “abortion ban*”[tiab] OR “abortion restriction*”[tiab] OR “trigger law*”[tiab] OR “Texas SB 8”[tiab] OR SB8[tiab]) AND (“Electronic Health Records”[Mesh] OR “electronic health record*”[tiab] OR EHR[tiab] OR “health information exchange”[tiab] OR HIE[tiab] OR HIPAA[tiab] OR “protected health information”[tiab] OR privacy[tiab] OR subpoena*[tiab] OR “law enforcement”[tiab]) AND (PPROM[tiab] OR “premature rupture of membranes”[tiab] OR hemorrhag*[tiab] OR miscarriage[tiab] OR “pregnancy loss”[tiab] OR sepsis[tiab] OR “septic abortion”[tiab] OR “uterine evacuation”[tiab] OR “time to intervention”[tiab] OR counseling[tiab] OR “informed consent”[tiab] OR documentation[tiab])

Full database-specific search strings are provided in the Appendices. For Google Scholar, results were screened using relevance sorting, and the first ~200 results per query were reviewed, consistent with scoping review conventions.

All retrieved records were exported into EndNote and deduplicated (Table 1). Of 263 total records, 93 duplicates were removed, leaving 170 unique records for title/abstract screening. Titles and abstracts were screened for relevance to post-*Dobbs* reproductive health surveillance/data pipelines and to downstream documentation or E-OB care effects. Full texts were obtained for all 170 records and assessed for eligibility when abstracts suggested relevance or when uncertainty remained. Given the interdisciplinary scope and scoping (rather than evaluative) aim of the review, a single reviewer conducted screening using conservative inclusion criteria, retaining ambiguous cases because the objective was mechanism identification rather than effect-size estimation. Reasons for full-text exclusion are provided in the Appendices. 

**Table 1:** Database yields before deduplication.

Source	Records identified
PubMed/MEDLINE	114
OpenAlex	17
HeinOnline (law/policy)	92
Google Scholar (hand-search)	40
Total before deduplication	263

**Figure 1:**
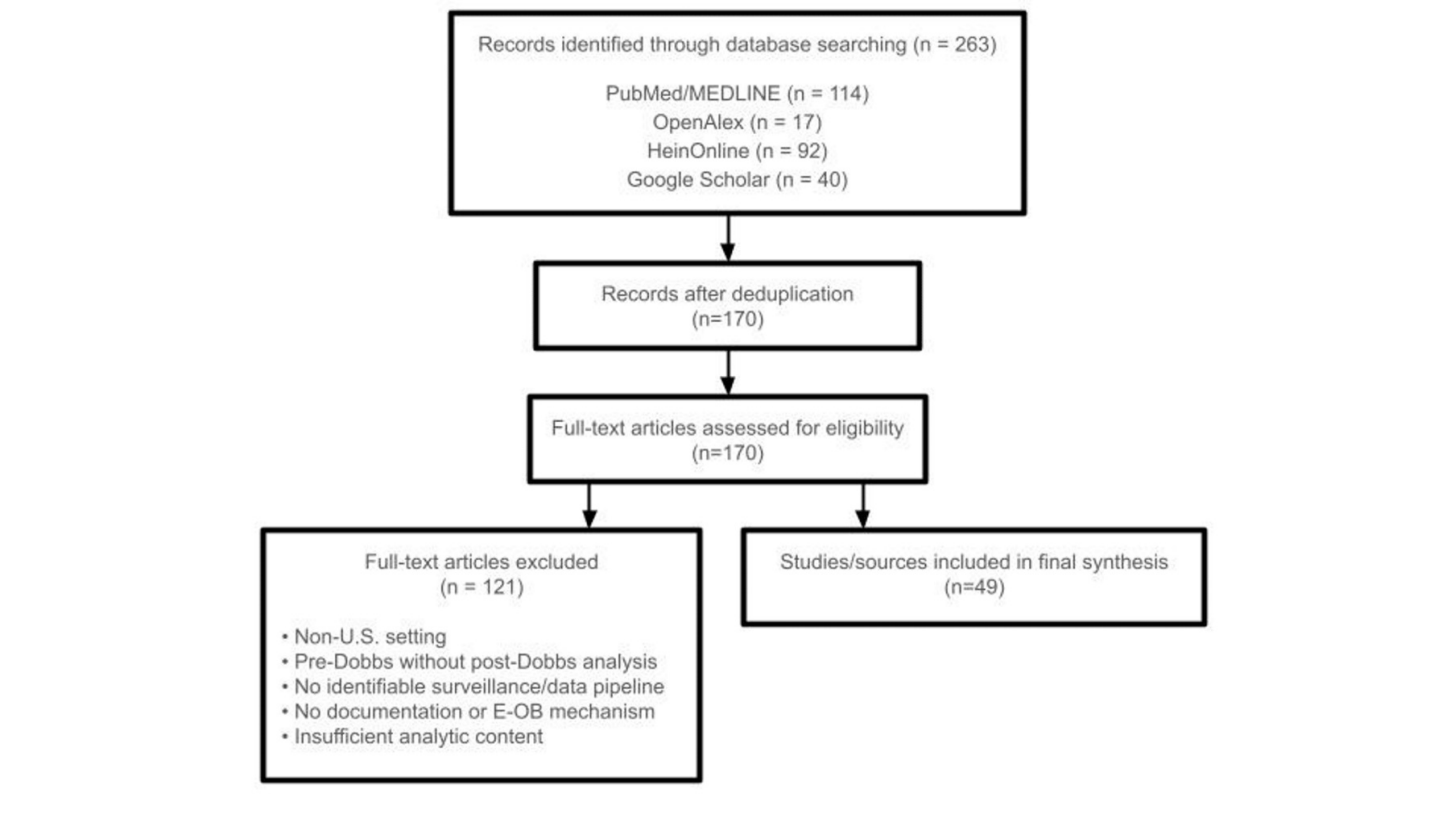
A PRISMA-ScR flow diagram summarizing study selection. PRISMA-ScR: Systematic Reviews and Meta-Analyses Extension for Scoping Reviews, E-OB: emergency obstetric.

Of the 170 full-text articles assessed, 121 were excluded (e.g., non-U.S. setting, pre-*Dobbs* without post-*Dobbs* analysis, no identifiable surveillance/data pipeline, no documentation/E-OB mechanism, or insufficient analytic content). Forty-nine (49) studies/sources met the inclusion criteria and were included in the final synthesis (Figure 1). Screening was completed on October 22, 2025.

Eligibility criteria required literature to document the link between (1) a data/surveillance pipeline (EHR/HIE/HIPAA, pharmacy/claims, apps/CPCs, or law-enforcement tools such as geofence/keyword warrants/subpoenas) and (2) a mechanism affecting practice (documentation changes, counseling/disclosure chill, or delay/time-to-intervention leading to E-OB signals such as hemorrhage, sepsis, or ectopic care).

The collected sources were categorized by both evidentiary strength and primary analytic contribution. Tier C included analytic, legal, and theoretical sources that provided foundational framing on HIPAA deficiencies, surveillance pipelines, legal epidemiology, and survey evidence on provider attitudes and public perceptions. Tier B included organizational and policy-operational sources documenting institutional risk-mitigation practices, such as revisions to release-of-information policies, the use of DUAs, and implementation of EHR workflow changes to manage interoperability risks. Tier A included operational clinical evidence, defined as quantifiable evidence of clinical impact, including measured changes in documentation practices (e.g., GCs using vague language, NM minimizing RHI collection) or delays in time-to-intervention for E-OB conditions (e.g., physician-reported ectopic delay data, PPROM outcome data).

The tiered framework is intended to identify plausible and documented pathways of ethical and clinical risk rather than to assert linear causality. The evidence demonstrates convergence across tiers, not definitive attribution of outcomes to singular mechanisms. This interdisciplinary approach, integrating legal epidemiology, qualitative narratives, and clinical data, was necessary to capture the scope of post-*Dobbs* impacts across healthcare and criminal justice systems. A formal risk-of-bias assessment was not performed because this study was designed as a scoping review focused on mapping concepts and mechanisms rather than evaluating effect sizes, consistent with the PRISMA-ScR methodology.

Results

Across the included studies, results converged around four primary domains: (1) surveillance and legal exposure, (2) changes in documentation and clinical communication, (3) delays in E-OB intervention, and (4) disproportionate impacts on marginalized populations.

Evidence Synthesis and Systems Map 

**Figure 2:**
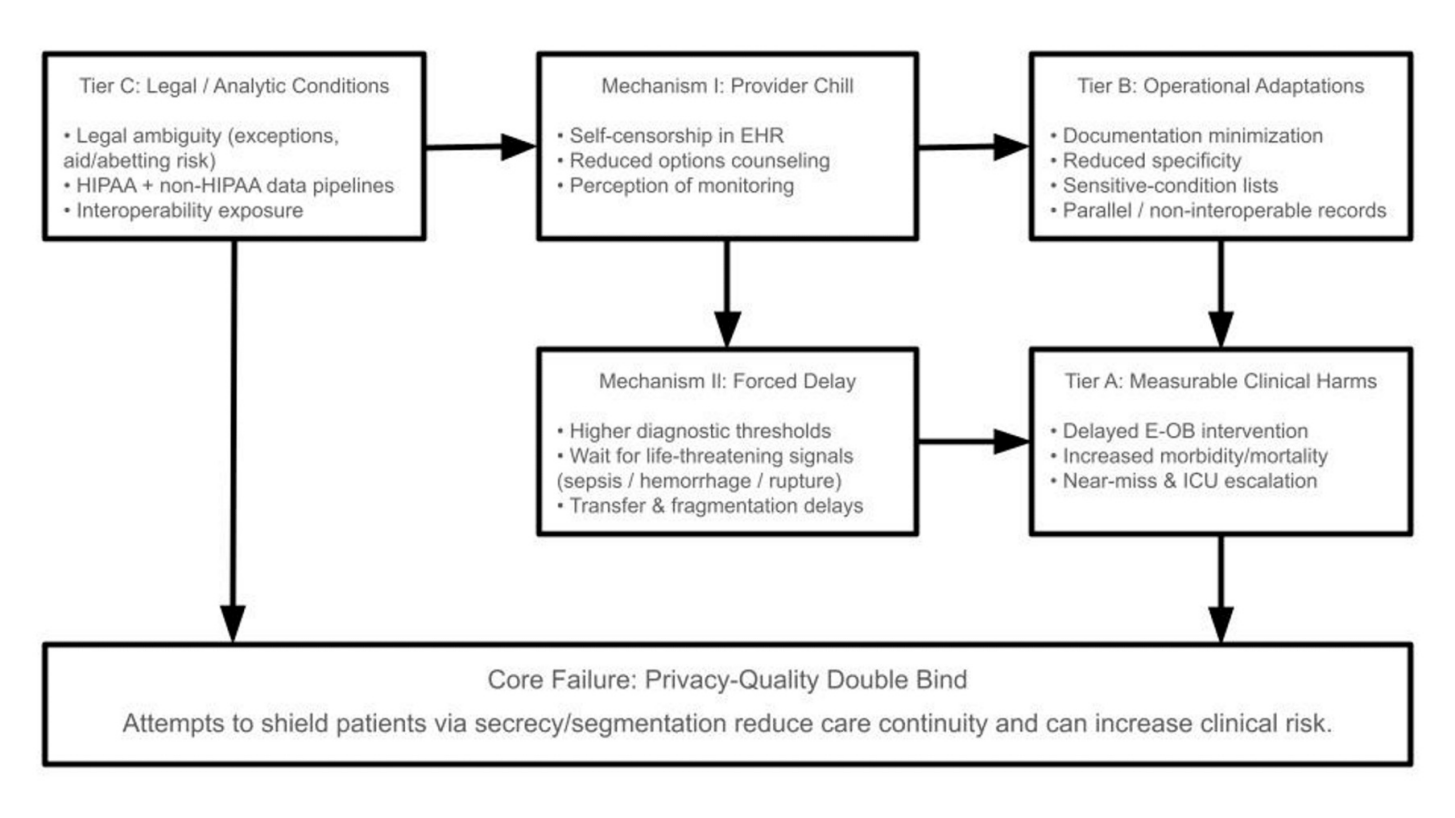
Tiered pipeline-to-harm model. HIPAA: Health Insurance Portability and Accountability Act, EHR: electronic health record, E-OB: emergency obstetric, ICU: intensive care unit.

Findings across the three evidence tiers are summarized in Figure 2 (Tiered pipeline-to-harm model) and Table 2 (Evidence tiers with representative sources), which together map how legal-analytic conditions (Tier C) drive organizational chaos (Tier B) and culminate in measurable clinical harm (Tier A).

**Table 2:** Tiered evidence structure as a synthesis heuristic. HIPAA: Health Insurance Portability and Accountability Act, E-OB: emergency obstetric, EHR: electronic health record, GCs: genetic counselors, NM: nurse-midwifery, RHI: reproductive health information, EDs: emergency departments, ICU: intensive care unit.

Tier (source type)	Input/cause	Process (mechanism I: documentation/chill)	Output (mechanism II: E-OB delay/harm)
C - Legal/Analytic	Legal Ambiguity (unclear exceptions, aid/abetting clauses); Pervasive Pipelines (HIPAA weakness, non-HIPAA digital data, warrants) [17].	Provider Chill: Fear leads to self-censorship, avoidance of legal options counseling, and the perception of a “politician in the room” [10].	Forced Delay: Physicians may defer intervention until patients develop life-threatening E-OB signals [39].
B - Organizational/ Policy	Interoperability Risk (EHR data accessible everywhere, cloud storage vulnerability) [3].	Documentation Restructuring: Institutions advise reducing specificity, creating sensitive condition lists, and potentially using parallel, noninteroperable records [3].	Fragmentation of Care: Increased patient transfers between systems due to divergent institutional policies, resulting in logistical hurdles and disrupted continuity [7].
A – Operational/ Clinical	Legal Fear/Risk of Enforcement (measured professional concern) [13].	Measured Practice Changes: GCs use vague language/code words [27]. NM departments reduce RHI collection [30]. EDs impose higher diagnostic thresholds [35].	Maternal Morbidity/Mortality: Measurable outcomes include 57% serious maternal morbidity with expectant management, delayed ectopic treatment, and cases escalating to sepsis/ICU admission due to delayed induction [40].

The core systemic malfunction is the Privacy-Quality Double Bind: efforts to protect patients from legal pipelines (Tier C) via organizational secrecy measures (Tier B, e.g., data segmentation or parallel records) inherently compromise information flow needed for integrated, high-quality care, driving the documented delays and morbidity (A-tier) [6]. The system sacrifices safety to achieve a fragile, legally contested form of privacy.

Discussion: slow harm in clinical practice

The reviewed evidence supports "slow harm" as a policy- and infrastructure-mediated consequence of post-*Dobbs *legislation. Physicians operating under vague state laws and limited institutional guidance may increasingly treat time-sensitive E-OB conditions as potential legal risks. Waiting until a patient with PPROM develops sepsis or hemorrhage before intervening, or requiring additional, non-standard imaging and labs to reach a higher threshold of certainty for ectopic diagnosis, may contribute to deviations from evidence-based care [36]. These delays raise not only questions of legality but of professional ethical breach, as clinicians are structurally compelled to violate standards of beneficence and nonmaleficence under threat of legal sanction. Defensive shifts-such as GCs making records vague or NM departments minimizing RHI collection-compromise the integrity of the medical record, introducing long-term risks to care continuity, research, and public health surveillance [27,29].

After *Dobbs*, federal and state privacy policy shifts have not resolved this bind and remain unstable. In April 2024, HHS finalized the HIPAA “Reproductive Health Care Privacy Rule,” which would have narrowed PHI disclosure by prohibiting releases for investigations or liability tied to lawful reproductive care and by requiring signed attestations for certain requests [41]. But in June 2025, a federal court vacated most of the rule nationwide, and subsequent appellate action left enforcement largely nullified, reopening disclosure risk and extending institutional ambiguity [42]. Meanwhile, state consumer-health privacy laws such as Washington’s My Health, My Data Act partially address non-HIPAA gaps affecting FemTech apps and data brokers, yet protections remain patchwork and jurisdiction-dependent [43]. As a result, clinicians and health systems continue to operate under a moving legal target, reinforcing documentation chill and delay mechanisms.

Mitigation strategies

Effective mitigation requires simultaneous efforts to harden data infrastructure (Tier B), restore clinical autonomy (Tier A), and pursue longer-term legal reform (Tier C). At the operational level, institutions can reduce exposure by defining safer data and care workflows [3]. This includes reviewing server locations, hosting data in less restrictive jurisdictions where feasible, treating reproductive care information as highly protected within EHRs, and blocking automatic data sharing [3]. Clinicians may also benefit from system-level supports: pediatric PCPs, for example, have endorsed EMR-integrated clinical decision support (69%) and standardized order sets (57%) as tools to reduce paralysis and support consistent care [24]. Where full telehealth abortion is infeasible, partial telehealth “unbundling” may allow screening, counseling, and follow-up to occur remotely, reducing risk while supporting brick-and-mortar services [32]. Institutions can further reduce exposure through DUAs requiring business associates to follow internal policies and to provide notice before releasing records to law enforcement, although strategies such as out-of-pocket payments to avoid claims trails must be evaluated against their equity implications [34].

Longer-term safety requires systemic changes to data privacy law. Strengthening HIPAA to limit law-enforcement access to RHI and considering heightened privacy standards analogous to those applied to psychotherapy notes or substance use disorder records would address part of the problem [5]. However, durable reform must also reach consumer data ecosystems by addressing apps, data brokers, and surveillance tools such as geofence warrants through broader federal privacy legislation [9]. Regulatory gaps could also be narrowed by treating reproductive-data applications as covered entities and reclassifying Software Applications for Contraception (SACs) under the FDA as Class III high-risk medical devices (such as IUDs (intrauterine devices)) to increase premarket review, auditing, and ongoing oversight [18]. These reforms should be paired with design principles that avoid secrecy-based workarounds that fragment records and create care risks [3]. Stronger privacy protections are likewise necessary to protect research integrity. With roughly 40% of women reportedly unwilling to participate in studies requiring daily menstrual tracking, app-based research needs stronger protections [44]. Without safeguards, surveillance fears will erode participation, bias data toward lower-risk populations, and impede women’s health research [44].

Finally, clinical mitigation must also reduce knowledge and comfort deficits that drive delay. Training should be expanded in emergency pregnancy options counseling, referrals, and, where legally permitted, medication abortion care [15]. Skills-based training, including manual uterine aspiration for miscarriage management and obstetric complications, should similarly be scaled to reduce delays tied to operating-room dependence [15].

Limitations

The current body of literature on *Dobbs*-related impacts is constrained by several factors inherent to the immediate post-ruling environment. Much clinical-harm evidence relies on provider-submitted narratives and preliminary qualitative findings, such as the Care Post-Roe study and physician interviews, which limit estimates of how often care deviates from standard practice [7]​​​​​. Survey data, particularly from emergency physicians, may also reflect selection bias, overrepresenting clinicians with stronger views or greater exposure to these issues [15]. Because enforcement and litigation have been most visible in a small number of highly restrictive states (e.g., Texas), the empirical and narrative corpus may be geographically skewed, undercapturing slower or less publicized harms elsewhere. In addition, race- and ethnicity-stratified data remain limited or inconsistently reported across post-*Dobbs* studies, constraining analysis of differential impacts and obscuring how documentation practices and delays may disproportionately affect marginalized patients. Additionally, the use of a single reviewer for study selection may introduce selection bias, which is a recognized limitation of scoping review methodology.

The rapidity and inter-jurisdictional evolution of state laws produce a “regulatory fog” that complicates both research and institutional compliance [16]​​​​​​. Finally, because much of the literature focuses on provider perspectives, future work must foreground patient experiences to fully characterize barriers and harms [10].

Future directions

Future research should quantify the long-term incidence and scope of slow harm by linking policy changes to maternal morbidity and mortality outcomes [45]. Work is needed to quantify trends in access to, and potential coercion toward, contraception and sterilization across differing policy environments, particularly among marginalized populations [45].

Given the reliance on digital mitigation, research must evaluate the efficacy of current protective measures, such as the accuracy and adoption of EHR data segmentation standards (DS4P) in HIEs [5]. Continued work should also examine the ethical and operational impacts of legal uncertainty on maternal-fetal research, which faces chilling effects on data collection and IRB processes in restricted states [46]. Adopting a reproductive justice lens will be crucial to ensuring that policy solutions prioritize communities at the highest risk of legal and health consequences and address intersecting systemic harms [1]​​​​​​.

## Conclusions

*Dobbs *represents a profound legal and technological disruption, challenging the assumption that medical practice is both legal and confidential. The post-*Dobbs* environment has increasingly transformed data and surveillance pipelines into potential sources of legal exposure for patients and providers. These conditions contribute to forms of “slow harm” characterized by documentation and communication chill, while operational evidence suggests dangerous delays in treating critical conditions such as ectopic pregnancy and PPROM-related complications. Across legal, organizational, and clinical domains, the reviewed literature consistently identified patterns of fragmented communication, defensive documentation practices, and elevated thresholds for intervention in time-sensitive obstetric care. To safeguard patient autonomy, prevent further preventable morbidity and mortality, and address inequitable consequences falling on marginalized communities, reforms must confront both technological vulnerabilities in data systems and the systemic chaos driving clinical delays. The crisis presents an urgent opportunity to rebuild a more equitable, privacy-protective reproductive healthcare infrastructure.

## Appendices

**Table 3:** PRISMA-ScR flow template. PRISMA-ScR:  Systematic Reviews and Meta-Analyses Extension for Scoping Reviews.

Stage	Count	Notes	Query
Records identified (PubMed)	114		
	5		(("Dobbs"[tiab] OR "post-Roe"[tiab] OR "abortion ban*"[tiab] OR "abortion restriction*"[tiab] OR "trigger law*"[tiab] OR "Texas SB 8"[tiab] OR SB8[tiab]) AND ("Electronic Health Records"[Mesh] OR "electronic health record*"[tiab] OR EHR[tiab] OR "health information exchange"[tiab] OR HIE[tiab] OR HIPAA[tiab] OR "protected health information"[tiab] OR privacy[tiab] OR subpoena*[tiab] OR "law enforcement"[tiab]) AND (PPROM[tiab] OR "premature rupture of membranes"[tiab] OR hemorrhag*[tiab] OR miscarriage[tiab] OR "pregnancy loss"[tiab] OR sepsis[tiab] OR "septic abortion"[tiab] OR "uterine evacuation"[tiab] OR "time to intervention"[tiab] OR counseling[tiab] OR "informed consent"[tiab] OR documentation[tiab]))
	74		(("Dobbs"[tiab] OR abortion[tiab] OR "post-Roe"[tiab]) AND (HIPAA[tiab] OR "Health Insurance Portability and Accountability Act"[tiab] OR "protected health information"[tiab] OR "health information exchange"[tiab] OR EHR[tiab] OR HIE[tiab] OR privacy[tiab]) AND ("reproductive health"[tiab] OR pregnan*[tiab] OR obstetric*[tiab]))
	33		(("Dobbs"[tiab] OR "post-Roe"[tiab] OR "abortion ban*"[tiab] OR "abortion restriction*"[tiab]) AND (documentation[tiab] OR "medical record*"[tiab] OR "clinical decision-making"[tiab] OR counseling[tiab] OR "informed consent"[tiab]) AND (pregnan*[tiab] OR obstetric*[tiab] OR miscarriage[tiab] OR PPROM[tiab]))
Records identified (Open Alex)	17		
	0		("keyword warrant*" OR "reverse keyword") AND (abortion OR reproductive)
	0		(("geofence" OR "reverse location") AND warrant*) AND (abortion OR reproductive)
	1		("automated license plate" OR ALPR OR ANPR OR "license plate reader*") AND (abortion OR reproductive OR "health data" OR "medical record*")
	16	articles only	(Dobbs OR "post-Roe" OR "abortion ban*" OR "abortion restriction*" OR "trigger law*" OR "Texas SB 8" OR SB8 OR EMTALA) AND (HIPAA OR "protected health information" OR "electronic health record*" OR EHR OR "health information exchange" OR HIE OR surveillance OR "pharmacy record*" OR "period app*" OR "menstrual app*" OR "crisis pregnancy center*" OR CPC OR subpoena* OR "law enforcement") AND (PPROM OR "preterm premature rupture of membranes" OR "prelabor rupture of membranes" OR hemorrhag* OR miscarriage OR "pregnancy loss" OR sepsis OR "septic abortion" OR "uterine evacuation" OR ("time" NEAR/3 intervention) OR counseling OR "informed consent" OR documentation OR "medical record*")
Records identified (HeinOnline / law-policy)	92		below queries were hand-searched for relevance
	120		("geofence" OR "reverse location") AND (abortion OR reproductive)
	32		("keyword warrant" OR "reverse keyword") AND (abortion OR reproductive)
	110		("automated license plate" OR ALPR OR ANPR) AND (abortion OR reproductive)
	276		("health information exchange" OR HIE) AND (abortion OR reproductive)
	2571		("crisis pregnancy center" OR CPC) AND (privacy OR data)
	653		(HIPAA OR "protected health information") AND (reproductive OR abortion) AND (disclosure OR subpoena OR attestation)
Records identified (Google Scholar)	40		hand-search
Total records before deduplication	263		
Duplicates removed	93		
Records after deduplication	170		
Full texts assessed for eligibility	170		
Full texts excluded, with reasons	121		
Studies/sources included in synthesis	49		

**Table 4:** Screening tracker.

Record_ID	Title	Year	Journal/source	Authors	DOI/URL	Database	Date_Imported	US_PostDobbs (Y/N/Unsure)	Pipeline_present (EHR/HIE/HIPAA/Pharmacy/Apps/CPC/ALPR/Geofence/Keyword)	Pipeline_flag (Y/N)	Mechanism_present (Counseling/Documentation/Approvals/Delay)	Mechanism_flag (Y/N)	Emergency_OB_focus (PPROM/Hemorrhage/Miscarriage/Sepsis/etc.)	Emergency_OB_flag (Y/N)	Decision (Y/Maybe/N)	Exclusion_reason (if N)	Notes (1-line why/what)	Date_Decision
House2022	What's Next: The Threat to Individual Freedoms in a Post-Roe World: Hearing Before the Committee on the Judiciary, House of Representatives, One Hundred Seventeenth Congress, Second Session	2022					10/7/25								N	not empirical	Congressional hearing; background only	10/7/25
Adashi2025	The Privacy of Reproductive Health Care Data: A Critical Health Insurance Portability and Accountability Act of 1996 Update	2025	J Womens Health	Adashi, E.Y., O'Mahony, D.P. & Cohen, I.G.	DOI: 10.1089/jwh.2025.0036	PubMed	10/7/25	Y	HIPAA/PHI/Claims, Apps/digital data	Y	approvals/attestations, documentation changes, delay/access	Y		N	Y		Analyzes the HIPAA Final Rule issued in April 2024; focuses on data privacy policy, but discusses mechanisms protecting all reproductive healthcare data, which includes E-OB care	10/21/25
Ahmmed2022	Sexual and reproductive health experiences of adolescent girls and women in marginalised communities in Bangladesh	2022		Ahmmed, F., Chowdhury, M. S., Helal, S. M.			10/7/25									not US	not US; Bangladesh SRH experiences	10/7/25
Allison2025	Exploring primary care physician biases in adolescent contraceptive counseling	2025		Allison, B. A. Bullington, B. W. Makhijani, S. A. Arora, K. S.			10/7/25								N	no pipeline / not emergency OB	PCP bias in adolescent contraceptive counseling. Not emergency OB	10/7/25
Allyse2022	Prenatal genetics in a post-Roe United States	2022		Allyse, Megan, Michie, Marsha			10/7/25								N	No pipeline; no eempirical measures of documentation changes, approvals, or time-to-intervention, and no E-OB outcomes (PPROM, septic miscarriage, ectopic).	Conceptual analysis on prenatal testing, “reason bans,” early prenatal care avoidance; equity emphasis.	10/7/25
Annas2022	Trust, Brutality, and Human Dignity: How "Partial Birth Abortion" Helps Shape American Biopolitics	2022		Annas, G. J.			10/7/25								N	no pipeline	law/ethics essay; not clinical ops.	10/7/25
Arey2023	Abortion Access and Medically Complex Pregnancies Before and After Texas Senate Bill 8	2023	Obstet Gynecol	Arey, W., Lerma, K., Carpenter, E., Moayedi, G., Harper, L., Beasley, A., Ogburn, T. & White, K.	DOI: 10.1097/aog.0000000000005153	PubMed	10/7/25	Y	Documentation/Institutional Policies	Y	delay/counseling	Y	Medically Complex Pregnancies/Fetal Diagnoses	Y	Y	fits scope	TX SB8; evaluate how Texas health care professionals navigate abortion restrictions when managinf patients with medically complex pregnancies	10/21/25
Atkins2022	Over-the-counter provision of emergency contraceptive pills: a systematic review	2022		Atkins, K. Kennedy, C. E. Yeh, P. T. Narasimhan, M.			10/7/25								N	no pipeline / not emergency OB	Systematic review of OTC ECP; outside scope.	10/7/25
Auteri2023	The Ripple Effect of Dobbs on Geofence Warrants Symposium Issue: Notes	2023	N. Ky. L. Rev.	Auteri, S.		HeinOnline	10/7/25	Y	documentation/EHR risk, pharmacy/claims, law enforcement/surfeillance		Delay/time-to-intervention	Y	ectopic/miscarriage, morbidity/complications	Y	Y		Dobbs decision effects on delivery of medical care	18-Oct
Bach2023	HIPAA v. Dobbs	2023	Berkeley Tech. L.J.	Bach, W.A.T.		HeinOnline	10/7/25	Y	HIPAA/Apps/Geofence (Websites selling abortion pills sharing sensitive data; settlement prohibiting 'Geofencing' around facilities)	Y	Disclosure Chill/Documentation (Need to protect reproductive health data; legal risk)	Y		N	Y		Legal analysis covering HIPAA shortcomings and digital surveillance risks (pill websites sharing data, geofencing) post-Dobbs	18-Oct
Baker2023	Texas Senate Bill 8 and Abortion Experiences in Patients With Fetal Diagnoses: A Qualitative Analysis	2023	Obstet Gynecol	Baker, C.C., Smith, E., Creinin, M.D., Moayedi, G. & Chen, M.J.		PubMed	10/7/25	Y	documentation and institutional policies	Y	disclosure chill (documentation is restricted --> practice becomes more "back alley"	Y	medically Complex Pregnancies/Fetal Diagnoses	Y	Y	fits scope	Qualitative, TX SB8 fetal diagnoses; approvals/deferrals/delay.	18-Oct
Bakhtari-Aghdam2023	The effect of counseling on women's coping with unplanned pregnancy	2023		Bakhtari-Aghdam, F. Abedini, S. Nourizadeh, R. Mehrabi, E. Havizari, S. Nikkhesal, N.			10/7/25								N	not US	Counseling/coping study outside U.S.	10/7/25
Barrera2023	Protecting Reproductive Rights Post-Roe: Can Companies Keep Your Data Safe?	2023	Business and Human Rights Journal	Barrera, M. & Labrin, D.R.		Google Scholar	10/7/25								N	Insufficient pipeline linkage	Corporate/consumer data-safety post-Roe; policy only	10/7/25
Bathke2024	Google and the Role of Surveillance Intermediaries in Geofence Warrants Comments	2024	Tul. J. Tech. & Intell. Prop.	Bathke, B.		HeinOnline	10/7/25								N	Insufficient pipeline linkage	Surveillance intermediaries in geofence warrants	10/7/25
Bentz2023	Defend Our Data: A Call on Lawmakers to Strengthen Reproductive Healthcare Data Privacy after the Fall of Roe	2023		Bentz, Kristen			10/7/25								N	not empircal / advocacy	Call to lawmakers; background only	10/7/25
Bhandari2024	"We just need to create as many avenues for access as we possibly can": Clinician and administrator attitudes toward telehealth medication abortion in the U.S. South	2024	Contracept X	Bhandari, P., Narasimhan, S. & Newton-Levinson, A.	DOI: 10.1016/j.conx.2024.100112		10/7/25	Y	privacy/digital data risk, telehealth, law enforcement/litigation risk	Y	delay/time-to-intervention, counseling/disclosure chill	Y	ecctopic	Y	Y		Clinician/administrator telehealth attitude.	18-Oct
Biggs2024	Young People's Support for and Personal Interest in an Advance Provision Model for Medication Abortion	2024		Biggs, M. A. Ehrenreich, K. Morris, N. Bachrach, L. Crespin, J. Grossman, D.			10/7/25								N	no pipeline / not emergency OB	Advance-provision attitudes; access, not clinical decision-making.	10/7/25
Blaylock2022	Client perspectives on choice of abortion method in England and Wales	2022		Blaylock, R., Makleff, S., Whitehouse, K. C., Lohr, P. A.			10/7/25	N							N	not US	England/Wales	10/7/25
Bornstein2022	Documenting activism and advocacy around medication abortion in Central, East, and West Africa	2022		Bornstein, M. Young, Y. Y. Pizzarossa, L. B. Cohen, M. J. Maina, J.			10/7/25								N	not US	Africa	10/7/25
Brandell2022	Telemedicine as an alternative way to access abortion in Italy and characteristics of requests during the COVID-19 pandemic	2022		Brandell, K. Vanbenschoten, H. Parachini, M. Gomperts, R. Gemzell-Danielsson, K.			10/7/25								N	not US	Italy, COVID-era	10/7/25
Brar2024	Navigating the Impact of the Dobbs Decision: Perspectives from Pediatric Surgeons on Reproductive Healthcare	2024	J Am Coll Surg	Brar, A., Mannava, S.V., Patwardhan, U.M., Sullins, V.F., Berdan, E.A., Greves, C.D., Gow, K.W., Carlisle, E., Tsao, K., Hunter, C., Baerg, J.E. & Knod, J.L.	DOI: 10.1097/xcs.0000000000001092	PubMed	10/7/25	Y	Legal speech/restriction pathway; not tech/data pipeline	Y	Chilled/coded prenatal counseling; narrowed informed consent; clinician moral distress	Y		N	Y		Pediatric surgeons describe counseling/code-switching and legal ambiguity after Dobbs; supports chilled-communication mechanism.	18-Oct
Braun2024	Safe Access Zone Legislation and Its Compliance with the Human Rights of Anti-Abortion Protesters in Australia	2024		Braun, K., Butcher, S.			10/7/25								N	not US	Australia protest law.	10/7/25
Brown2025	Documentation of Pregnancy Options Counseling in a Pediatric Emergency Department and Outpatient General Pediatric Clinic: A Single-Center Retrospective Study	2025	Clinical Pediatrics	Brown, M., Fitzgerald, H.N., Sola Rufai, S., Bell, L.A., Syed, T. & Kirkpatrick, L.	DOI: 10.1177/00099228251372395	PubMed	10/7/25								Y		Pediatric ED/clinic documentation of pregnancy-options counseling	18-Oct
Buchbinder2025	The Third Person in the Room	2025		Buchbinder, M.			10/7/25								N	not empirical	Ethics essay.	10/7/25
Buijsen2023	On Interpretation and Appreciation. A European Human Rights Perspective on Dobbs	2023		Buijsen, M.			10/7/25								N	not US	European human-rights perspective.	10/7/25
Bullington2025	Clinician Perspectives on Adolescent Contraceptive Counseling Following Dobbs v. Jackson: Implications for Young People's Contraceptive Autonomy	2025		Bullington, Brooke W. Mann, Emily S. Thornton, Madeline Hartheimer, Joline Arora, Kavita Shah Allison, Bianca A.			10/7/25								N	No clinical mechanism/outcome	Adolescent contraception counseling (access/education), not emergency-OB decision-making or data pipeline.	10/7/25
Callegari2025	Implementing digital sexual and reproductive health tools: Challenges and recommendations post-Dobbs	2025	Contraception	Callegari, L.S., Mosley, E.A., Borrero, S., Talabi, M.B., Kazmerski, T.M., Donley, G. & Krishnamurti, T.		PubMed	10/7/25	Y	EHR/HIPAA/Apps (patient-facing SRH apps integrated with EHRs; HIPAA reproductive privacy rule discussed)	Y	Counseling, Documentation, Delay/Approvals (privacy concerns constrain documentation, counseling, research delays)	Y		N	Y	Apps/CPC pipeline	Digital SRH tools post-Dobbs; keep if it covers provider workflows, privacy/disclosure, or EHR integration.	18-Oct
Campbell2024	Post-Dobbs Challenges in Research and Patient Protections	2024	JAMA psychiatry	Campbell, A.N.C. & Greenfield, S.F.		Google Scholar	10/7/25	Y	HIPAA (research PHI/confidentiality; subpoenas/Certificates of Confidentiality implications).	Y	chilled/cautious documentation and data-handling; participation/recruitment impacts.	Y		N	N	Policy/informatics	Patient protections/HIPAA/IRB challenges post-Dobbs	10/7/25
Canepa2025	How abortion restrictions compromise progress in interventional maternal‐fetal research	2025	Obstetrics & Gynecology	Canepa, E., Freedman, L., Talati, A., Sparks, T., Hodoglugil, N., Gonzalez‐Velez, J. & Brown, J.	DOI: 10.1002/pmf2.70095	OpenAlex	10/7/25	Y	ALPR / cross-state law-enforcement monitoring; institutional legal review for abortion-related car	Y	Delays in managing procedure-related complications; consent/counseling constrained by law; potential transfers/relocation	Y			Y	Policy/research ops	Shows Dobbs-era restrictions forcing delay and travel for maternal–fetal trial complications; includes ALPR surveillance example. It talks about researchers having to rewrite consent, secure funding for relocation, or even pause trials because they can’t guarantee abortion if a complication happens.	18-Oct
Casas2022	Managing Undesired Pregnancy After Dobbs	2022		Casas, R. S. Horvath, S. K. Schwarz, E. B. Bachorik, A. E. Chuang, C. C.			10/7/25								N	not empirical	JGIM commentary; no operational/empirical content.	10/7/25
Catarozoli2025	A Post-Dobbs World: More than One Right to Privacy under Attack	2025		Catarozoli, Sarah Jane			10/7/25								N	not empirical	Law review privacy broad essay; background only.	10/7/25
Cespedes2023	Uncharted Boundaries: Exploring Geofence Warrants as an Investigative Tool in Abortion-Related Criminal Investigations Post-Roe	2023		Cespedes, Denise			10/7/25								N	not US	Ethiopia.	10/7/25
Chekol2023	Person-centered abortion care in public health facilities across four regions of Ethiopia: a cross-sectional quantitative study of client experiences	2023		Chekol, B. M. McCaffrey, S. Dijkerman, S. Acre, V. Biru, D. D. Mehary, A. B. Muluye, S.			10/7/25								N	not US	Non-U.S. student SRH experiences.	10/7/25
Citron2023	Intimate privacy in a post-Roe world	2023	Fla. L. Rev.	Citron, D.K.		Google Scholar	10/7/25	Y	Apps / Geofence (location data to clinics) / Keyword-style ad trail	Y		N		N	N	policy/informatics. Pipeline-only article	Post-Dobbs U.S. law-review piece showing how existing app/location/data-broker pipelines can be weaponized against abortion seekers, helpers, and providers; strong policy/remedy value.	10/7/25
Clark2024	Consumer Privacy and the Dobbs Disruption	2024	U. Mich. JL Reform	Clark, M.R.		Google Scholar	10/7/25	Y	Consumer/app/rideshare/hotel data + CPCs not covered by HIPAA → available to AGs/LE; warns of activists using state privacy regimes to get data.	Y	warns that gaps in state consumer privacy laws can lead to chilling, abusive privacy requests, nuisance litigation, and selective enforcement	Y?		N	Y	policy/informatics	Consumer privacy + Dobbs. Good background on states’ consumer privacy gaps after Dobbs (esp. CPCs not under HIPAA + MHMDA limits) but doesn’t show practice-level effect	18-Oct
Clayton2022	Dobbs and the future of health data privacy for patients and healthcare organizations	2022	J Am Med Inform Assoc	Clayton, E.W., Embí, P.J. & Malin, B.A.	DOI: 10.1093/jamia/ocac155Link to doi.org	PubMed	10/7/25	Y	HIPAA/Apps/CPCs (HIPAA disclosure rules; mobile health app industry; CPCs collecting data)	Y	Delay/Counseling Chill (Confusion post-Roe spurs delays/denials for life-saving pregnancy care; legal risks lead to physicians facing difficult choices)	Y	Miscarriage/Ectopic (Confusion spurs delays/denials for miscarriage and ectopic pregnancy care)	Y	Y	Policy/informatics _ documentation	Perspective on HIPAA limitations, warning that data collection by apps/CPCs creates legal risk and contributes to care delays for emergent conditions	18-Oct
Cleeve2024	No test medical abortion - a review of the evidence on selective use of preabortion testing	2024		Cleeve, A. Wallengren, E. Brandell, K. Lee, S. Endler, M. Reynolds-Wright, J.			10/7/25								N	not emergency OB	Preabortion testing review; not focused on clinical decision-making in emergencies.	10/7/25
Constant2022	Could routine pregnancy self-testing facilitate earlier recognition of unintended pregnancy? A feasibility study among South African women	2022		Constant, D. Lopes, S. Grossman, D.			10/7/25								N	not US	South Africa feasibility study.	10/7/25
Cui2023	Why women choose self-managed telemedicine abortion in the Netherlands during the COVID-19 pandemic: a national mixed methods study	2023		Cui, N. Gemzell-Danielsson, K. Gomperts, R.			10/7/25								N	not US	Netherlands telemed.	10/7/25
Culter2025	Experiences of obstetrician-gynecologists providing pregnancy care after Dobbs	2025	JAMA Network Open	Cutler, A.S., Hale, C.M., Bennett, E., Jacques, L. & Higgins, J.		PubMed	10/7/25	Y	Institutional/Transfers (Institutional roles shaping physician experiences; transfers of care)	Y	Delay/Time-to-Intervention/Counseling Chill (Providers practicing under the threat of criminalization; uncertainty and confusion in care provision)	Y	Ectopic/PPROM (References clinical guidelines for ectopic pregnancy and prelabor rupture of membranes (PPROM))	Y	Y	fits scope (clinician mechanisms)	OB-GYNs describing uncertainty, institutional constraints, and implications for managing complications like ectopic pregnancy	18-Oct
Dang2022	Network pharmacological analysis and molecular docking of Huangqin-Baizhu herb pair in the treatment of threatened abortion	2022		Dang, C. X. Wang, D. Liu, P. F. Liu, J. X. Yu, X.			10/7/25								N	not relevant	Herbal docking study; unrelated.	10/7/25
DeAnna Baumle2023	Legal and Health Risks of Abortion Criminalization: State Policy Responses in the Immediate Aftermath of Dobbs	2023	Journal of Law and Health (Vol. 37 No. 1)	DeAnna Baumle, J.D.		Google Scholar	10/7/25	Y	devotes a section (“State Laws Addressing Abortion Data Privacy Post-Dobbs”) to HIPAA gaps, data collection, and digital surveillance—explicitly linking reproductive-health data systems and law-enforcement access	Y	details how legal uncertainty and penalties cause documentation and counseling chill, hospital risk-averse policies, and care delays (e.g., Texas women denied treatment for pregnancy complications)	Y	delayed care for pregnancy complications, miscarriages, and lawsuits from patients denied timely intervention	Y	Y		Post-Dobbs state responses	18-Oct
Dellinger2024	Bodies of evidence: The criminalization of abortion and surveillance of women in a post-Dobbs world	2024	Duke Journal of Constitutional Law & Public Policy	Dellinger, J. & Pell, S.		Google Scholar	10/7/25	Y	Law-enforcement surveillance tools (cell-site and GPS data, geofence warrants, ALPR, data brokers, social media scraping, digital search history)	Y	Documentation chill / risk aversion / delayed care (clinicians withholding care, requiring legal review before treatment; surveillance chills medical decision-making)	Y	Mentions pregnancy complications, miscarriage investigations, and delayed/denied care	Y	Y		Surveillance & criminalization	18-Oct
Dethier2025	Self-performed Rh typing: a cross-sectional study	2025		Dethier, D. Tschann, M. Roman, M. Chen, J. J. Soon, R. Kaneshiro, B.			10/7/25								N	not emergency OB	Self-performed Rh typing; outside scope.	10/7/25
Domingo2024	One Nation, under Dobbs: How Dobbs v. Jackson Women's Health Impacts Data Privacy for All	2024	UC L. Sci. & Tech. J.	Domingo, M.		HEIN	10/7/25	Y	EHR/HIE/HIPAA/Apps (examines EHR and data-sharing pipelines, HIPAA privacy gaps, HIE vulnerabilities, third-party apps and data brokers in post-Dobbs enforcement)	Y	Documentation & counseling chill / compliance uncertainty / legal review before care (providers fearful to record or share reproductive data; institutional delay pending legal interpretation)	Y	clinical hesitation around miscarriage and ectopic management	Y	Y	policy/informatics. Student-authored note. Framing of digital surveillance and CPCs is covered by Prince and Huq & Wexle	Data privacy after Dobbs	10/7/25
Dresser2023	Cruzan after Dobbs: What Remains of the Constitutional Right to Refuse Treatment?	2023		Dresser, R.			10/7/25								N	not empirical / no pipeline	Ethics essay on refusal rights; background only.	10/7/25
Drury2025	From Roe to Risk: The Sobering Realities of Reproductive Data Privacy in a Post-Roe America	2025	Journal of Health Care Law & Policy	Drury, B.		Google Scholar	10/7/25	Y	Apps / Data brokers / HIPAA gaps / Law-enforcement subpoenas (Meta warrants, period-tracking data)	Y	Documentation chill & disclosure fear (users and providers altering digital tracking, avoiding recordkeeping due to surveillance risk)	Y	Discusses miscarriage detection and abortion-related investigations (Nebraska case)	Y	N	Student-Authored Piece/Law Graduate. Overlaps significantly with core faculty articles on data brokers and period tracking apps.	Reproductive data privacy	10/7/25
Edeling2024	Phantom data and the potentials of radical caretaking in reproductive health	2024		Ebeling, M. F. E.			10/7/25								N	not empirical / thoery	Anthro essay; unlikely to have clinical ops.	10/7/25
Edouard2024	Value of self-care for sexual and reproductive health	2024		Edouard, L. Okonofua, F.			10/7/25								N	not US	Africa	10/7/25
Ehrenreich2023	Perspectives on Alternative Models of Medication Abortion Provision Among Abortion Patients in the United States	2023	Womens Health Issues	Ehrenreich K; Baba CF; Raif...		PubMed	10/7/25	unsure (Pre-Dobbs data 2017-2018; contextualized post-Dobbs 2023)	ocuses on telehealth, advance provision, and OTC models but not digital surveillance, EHR, or law-enforcement data pipelines	N	Access delay / travel burden / policy restriction effects (mentions long distances, multiple appointments, privacy concerns)	Y		N	N	No pipeline element present; study explores patient perspectives on access models pre-Dobbs, not surveillance or practice-chilling mechanisms.	Access/provision model	10/7/25
Envuladu2023	Healthcare workers' delivery of adolescent responsive sexual and reproductive healthcare services: an assessment in PL...	2023	BMC Womens Health	Envuladu EA; Massar K; ...			10/7/25	N		N		N		N	N	Not U.S.	Likely Nigeria/Plateau study; outside U.S.	10/7/25
Essien2025	The Dobbs Decision and Maternal Health: Using Real-World Data to Unveil the Effect on Abortion Access and Pregnancy...	2025	Obstetrics & Gynecology	Essien S; Kaur P; ...		OpenAlex	10/7/25	Y	EHR / HIE data platform (TriNetX federated electronic health record network)	Y	None explicit – study reports quantitative outcomes only, no mention of documentation changes, counseling, or delay mechanisms	N		N	N	Descriptive EHR-based outcome study; no discussion of practice mechanisms or clinical decision-making effects.	RWD focus	10/7/25
Falcone2024	The Evolution of Privacy Rights: Examining the Post-Dobbs Context	2024	Law review	Falcone DG			10/7/25	Y		N		N		N	N	Not empirical	General privacy essay; no operational detail	10/7/25
Feinberg2023	Healthcare and Privacy Law Consequences following Dobbs (Symposium intro)	2023	Journal of Law & Health	Feinberg R			10/7/25	Y		N		N		N	N	Not empirical	Symposium introduction; background only	10/7/25
Fetrow2024	Surveilling Pregnancy: Predictive Policing in a Post-Roe World	2024	UC L. SF J. Gender & ...	Fetrow SC		HeinOnline	10/7/25	Y	Predictive policing data integration; big-data aggregation from apps and digital communications; law-enforcement tools (ALPR, geofence warrants, data broker feeds)	Y		N		N	N	Conceptual/policy analysis only; no empirical evidence of pipeline use or mechanisms affecting clinical practice.	Theory-driven thesis on predictive policing; lacks real-world pipeline data or clinical mechanisms.	10/7/25
Fox2025	Does telemedicine hold the key for reproductive health care? A quantitative examination of women's intentions toward use	2025	Health Services Research	Fox G; Lynn T; van der Werff ...			10/7/25	Unsure		N		N		N	N	No clinical mechanism/outcome	Intentions to use telemedicine; not clinical decision-making or pipeline ops	10/7/25
Francis2024	Protecting Electronic Health Records after Dobbs	2024	Santa Clara Law Review	Francis LP		HeinOnline	10/7/25	Y	EHR/HIPAA/Law Enforcement Tools (State shield laws protect reproductive healthcare data; search warrants)	Y	Approvals/Documentation/Disclosure Chill (Proposed HIPAA attestation requirements; stopping state resources use to gain information)	Y		N	Y		Legal analysis addressing threats to EHRs post-Dobbs and state/federal efforts (HIPAA attestations, shield laws) to protect reproductive health data	10/7/25
Friend2025	Abortion AI: Toward an equity-centered research agenda for AI and abortion	2025	Contraception	Friend J; Brindis CD; Upadh...		PubMed	10/7/25	Y	AI/LLM/Digital Health/Law Enforcement Tools (AI tools tailored for abortion; ChatGPT; police obtaining Facebook messages)	Y	Counseling/Disclosure Chill (Digital privacy breaches endanger patient well-being; misinformation risk)	Y	Self-managed abortion	N	Y		Surveys AI implications for abortion, focusing on digital surveillance (Facebook data used in Nebraska case) and misinformation risks	18-Oct
Fulcher2022	Impact of the COVID-19 pandemic on abortion care utilization and disparities by age	2022	American Journal of Obstetrics & Gynecology	Fulcher IR; Onwuzurike C; ...			10/7/25	Unsure		N		N		N	N	No clinical mechanism/outcome	Pandemic-focused; not Dobbs/pipeline/clinical decision-making	10/7/25
Gawron2024	Family Planning for Patients With Inflammatory Bowel Disease in the Post-Dobbs Era	2024	Gastroenterology & Hepatology	Gawron LM; Johnson JB; ...			10/7/25	Y		N		N		N	N	Not emergency OB	Specialty perspective; outside emergency obstetric decision-making	10/7/25
Gentile2025	Evaluation of prenatal genetic counselors' abortion education and training as variables associated with self-efficacy	2025	Journal of Genetic Counseling	Gentile BJ; Araujo RL; ...			10/7/25	Y		N	Counseling (education/training)	Y		N	N	No relevant data pipeline	Education/self-efficacy study; lacks EHR/HIPAA/app or law-enforcement pipeline	10/7/25
Goodday2022	The Post-Roe Political Landscape Demands a Morality of Caution for Women's Health	2022	Journal of Medical Internet Research	Goodday S; Karlin D; Suver ...			10/7/25	Y		N		N		N	N	Not empirical	Editorial; lacks operational/clinical detail	10/7/25
Gosain2024	Dobbs in a Technologized World: Implications for US Data Privacy	2024	The Law and Ethics of Data	Gosain J; Keune JD		HeinOnline	10/7/25	Y	Apps/GPS/Financial Data/Law Enforcement Tools (Location data; money transfers; Facebook messages used to investigate)	Y	Disclosure Chill/Prosecution (Abortion privacy debate; how non-health data can infer secrets)	Y		N	Y		Analyzes data privacy implications, detailing surveillance via location data, financial records, and digital communications used to investigate abortion	18-Oct
Gosain2023	Dobbs in a Technologized World: Implications for US Data Privacy (update)	2023	The Law and Ethics of Data	Gosain J; Keune JD		HeinOnline	10/7/25	Y	EHR/HIPAA/Apps (policy)	Y		Y		N	N	Duplicate record	Appears to be an updated version; keep one canonical record after full-text check	10/7/25
Graf2023	Fetal anomaly diagnosis and termination of pregnancy	2023	Developmental Medicine & Child Neurology	Graf WD; Cohen BH; Kalsner L ...			10/7/25	Unsure		N		N		N	N	No clinical mechanism/outcome	General review; not Dobbs/pipeline-specific	10/7/25
Grossman2023	Care post-Roe: documenting poor-quality care related to abortion bans since the Dobbs decision	2023	Contraception	Grossman D; Joffe C; Kaller S; ...		PubMed	10/7/25	Y	EHR/Documentation/Transfers	Y	Delay/time-to-intervention	Y	Ectopic Pregnancy/Fetal Anomalies/Miscarriage	Y	Y		Direct qualitative documentation of substandard emergency care mechanisms post-Dobbs	10/7/25
Grunebaum2023	Counseling for the option of termination of pregnancy for severe fetal anomalies in light of recent Supreme Court ruling	2023	Seminars in Fetal & Neonatal Medicine	Grünebaum, A., Moreno, J. D., Esq, S. P., Chervenak, F. A.		PubMed	10/7/25	N	None – discussion centers on ethical counseling and state laws, not digital or data-surveillance infrastructures	Y	addresses counseling and disclosure chill under abortion bans (providers balancing ethical duties vs. criminal liability; altered counseling practices, civil-disobedience debate)	Y	severe fetal anomalies and perinatal outcomes (anencephaly, trisomy 13/18, renal agenesis, hydrops fetalis)	Y	Y		Ethical review of how state bans affect counseling and management of pregnancies with severe fetal anomalies; meets mechanism + E-OB criteria though lacks data pipeline.	18-Oct
Gupta2024	Rights-based reproductive services in medical schools in Rajasthan, Gujarat and Chandigarh, India: baseline findings	2024	Contraception & Reproductive Medicine	Gupta M; Iyengar K; Singla ...			10/7/25	N		N		N		N	N	Not U.S.	India study	10/7/25
Hall2023	Potential effects of Dobbs v. Jackson Women's Health on Civil Commitment Law	2023	American Journal of Law & Medicine	Hall TS		HeinOnline	10/7/25	Y		N		N		N	N	No relevant data pipeline	Civil commitment focus; not health-data or emergency OB	10/7/25
Hart2025	Before and after: The impact of the Roe v. Wade overturn on prenatal genetic counseling practice	2025	Journal of Genetic Counseling	Hart EJC; Dugan RB; He...		PubMed	10/7/25	Y	EHR/Documentation	Y	Counseling; Documentation Changes; delay	Y	fetal anomalies (study focuses on prenatal genetic counseling)	N	Maybe		Genetic counselors altered documentation (workarounds) and counseling practices due to legal uncertainty; patient travel time increased significantly	18-Oct
Hayry2023	Roe v. Wade and the Predatory State Interest in Protecting Future Cannon Fodder	2023	Cambridge Quarterly of Healthcare Ethics	Häyry, M.			10/7/25	Y		N		N		N	N	Not empirical	Philosophical essay	10/7/25
HendricksSturrup2023	An Assessment of Perspectives and Concerns Among Research Participants of Childbearing Age Regarding the Health-Related Data	2023	Journal of Medical Internet Research	Hendricks-Sturrup R; Lu CY		PubMed	10/7/25	Y	Apps/Search/Social Media/Credit Card (Google search history, online purchase history, text message data, credit card statements)	Y	Disclosure Chill/Prosecution (Concern about data security; lawyers used search activity and text message data in prosecution)	Y	Miscarriage/Stillbirth (References concerns about prosecution following miscarriage or stillbirth)	Y	Y		Survey detailing how digital footprints (search history, credit card data) are viewed as health-related information susceptible to misuse and prosecution post-Dobbs	18-Oct
Heuvel2023	Dobbsmacked by the Dobbs Decision: The Need for More Privacy Protection for Personal Health Information	2023	Mitchell Hamline Law Review	Vanden Heuvel M		HeinOnline	10/7/25	Y	HIPAA and provider-to-law-enforcement disclosure pipeline — extensive discussion of HIPAA Privacy Rule, HHS OCR guidance, and how PHI can reach prosecutors through subpoenas or hospital reporting	Y	Documentation / reporting / disclosure mechanisms — describes clinicians’ confusion, hospital risk-management policies compelling reporting, and resulting breaches of confidentiality	Y	Mentions miscarriage/stillbirth criminalization (e.g., Chelsea Becker case) but no clinical management or time-to-intervention data	Y	N	Student-Authored note. Legal discussion on HIPAA gaps and criminalization is covered more robustly by Bach & Terry and Tovino.	Legal analysis of HIPAA and provider reporting pipelines post-Dobbs; identifies hospital policy and documentation mechanisms enabling PHI transfer to law enforcement.	10/7/25
Hoang2024	Smile! You're on Camera: Police Use of Automatic License Plate Readers and the Need for Statewide Legislation	2024	University of Dayton Law Review	Hoang C		HeinOnline	10/7/25	Y	ALPR (Law-enforcement pipeline tool) – detailed analysis of police data capture, interstate data sharing, third-party vendors (Flock Safety), and lack of retention controls	N	None directly applicable – focuses on Fourth Amendment privacy and policy reform for law enforcement data rather than clinical practice	N		N	N	Outside scope – law enforcement data policy on ALPR, no clinical mechanism or E-OB focus	Legal review on ALPR data retention and privacy legislation; does not address reproductive health or clinical care mechanisms	10/7/25
Houser2023	" Guarding the Sanctity of Choice and Privacy": Data Privacy and Abortion-The next Frontier of the Fourth Amendment	2023	Northwestern Journal of Technology & Intellectual Property	Houser RS		Google Scholar	10/7/25	Y	Law-enforcement digital surveillance tools – detailed discussion of geofence warrants, keyword warrants, ALPR, cell-site location info, app data (Flo, Google, Facebook), and Stored Communications Act pipelines	Y	None directly; analyzes privacy violations and potential criminalization, not provider-facing practice mechanisms	N		N	N	Legal analysis of surveillance and Fourth-Amendment protections; no mechanism affecting medical documentation or care delivery	Focuses on state surveillance tools (geofence, search history, phone forensics) post-Dobbs; lacks provider-practice or patient-care mechanisms	10/7/25
Hovel2025	Inaction Despite Motivation: Assessing Systemic and Personal Barriers to Pediatricians' Post-Dobbs Emergency Contraception	2025	J Pediatr Adolesc Gynecol	Hovel E; Pickett M; Visotcky ...		PubMed	10/7/25	Y	EHR / EMR – mentions need for “clinical decision support built into the EMR” and “order set in the EMR” as system facilitators	Y	Documentation / workflow / counseling – surveys post-Dobbs provider behavior, low prescribing confidence, perceived barriers (“doesn’t occur to me,” “lack of clinic support”), and desired EMR-based QI mechanisms	Y		N	Y		Post-Dobbs survey identifying EHR/EMR pipeline gaps and practice mechanisms influencing EC provision among pediatric providers.	18-Oct
Howe2024	Ethical Risks of Systematic Menstrual Tracking in Sport	2024	J Bioeth Inq	Howe OR			10/7/25	Unsure	Apps (sport)	N		N		N	N	No clinical mechanism/outcome	Sport context; not emergency OB or clinical pipeline	10/7/25
Hsieh2022	Mifepristone (RU-486) as a Schedule IV Controlled Drug‚ÄîImplications for a Misleading Drug Policy on Women's Health	2022	Int J Environ Res Public Health	Hsieh YP; Wang YJ; Feng ...			10/7/25	Unsure		N		N		N	N	No clinical mechanism/outcome	Policy framing; not EHR/HIE/patient-level clinical decisions	10/7/25
Hunkler2025	Second Trimester Abortion: A Dilation and Evacuation Simulation for Gynecologic Surgery and Obstetrics Residents	2025	MedEdPORTAL	Hunkler K; Boedeker D ...			10/7/25	Y		N		N		N	N	Not emergency OB	Training simulation; not decision-making or data governance	10/7/25
Huq2023	Digital privacy for reproductive choice in the post-Roe era	2023	NYU Law Review	Huq AZ; Wexler R		HeinOnline	10/7/25	Y	EHR/Apps/PHI; subpoenas	Y	Counseling/ Disclosure Chill	Y		N	Y		Maps the bilateral economy of digital data flows (to patients and prosecutors) and proposes a new evidentiary privilege for reproductive choice.	10/7/25
Isaksen2022	Interviewing adolescent girls about sexual and reproductive health: a qualitative study exploring how best to ask questions	2022	Reproductive Health	Isaksen K; Sanday I; Zulu J ...			10/7/25	N		N		N		N	N	Not U.S.	Methods study; non-U.S.	10/7/25
Jackline2024	Exploring the utilization of postabortion care services at a tertiary facility in Gulu	2024	Womens Health (Lond)	Jackline A; Opee J; Bongomi ...			10/7/25	N		N		N	Postabortion care (non-U.S.)	N	N	Not U.S.	Uganda study	10/7/25
Jacques2023	"I'm going to be forced to have a baby": A study of COVID-19 abortion experiences on Reddit	2023	Perspect Sex Reprod Health	Jacques L; Valley T; Zhao S ...			10/7/25	Unsure		N		N		N	N	No clinical mechanism/outcome	COVID-era Reddit content; not clinical ops	10/7/25
Joseph2024	PRENATAL GENETIC COUNSELORS’CHANGING MEDICAL DOCUMENTATION PRACTICES IN THE AFTERMATH OF THE DOBBS V JACKSON SUPREME COURT DECISION	2024	Contraception	Joseph G; Harris-Wai J; Ridd ...		Google Scholar	10/7/25	Y	EHR/Documentation	Y	Documentation	Y		N	Y		Direct evidence of documentation changes post-Dobbs	10/7/25
Joubert2024	Impact of Roe v. Wade overturn on birth control prescriptions and sterilisation in rural North Carolina	2024	Eur J Contracept Reprod Health	Joubert E; Anderson T; Barre ...			10/7/25	Y		N		N		N	N	No clinical mechanism/outcome	Contraception/sterilization utilization; not emergency OB decision-making	10/7/25
Katznelson2025	An opportunity for guidance on reproductive health tracking technologies in a post-Roe United States	2025	Contraception	Katznelson G; McNamara I ...		PubMed	10/7/25	Y	Apps (Reproductive health tracking technologies)	Y	Disclosure Chill/Prosecution (References a Nebraska woman getting prison time for giving her daughter abortion pills)	Y		N	Y		Discusses the need for guidance on reproductive health tracking apps due to legal risks, citing a case involving prosecution for medication abortion	18-Oct
Kelly2025	Safeguarding autonomy: Implications of under-regulated period-tracking apps and paired devices	2025	Contraception	Kelly BG; Junchaya O; Min ...		PubMed	10/7/25	Y	Apps (Under-regulated period-tracking apps and paired devices)	Y	Disclosure Chill (Concerns about data privacy risk in the post-Roe environment; references weaponization of digital devices in criminal evidence)	Y		N	Y		Examines the implications of under-regulated period-tracking apps on user autonomy and privacy in the post-Roe landscape	18-Oct
Kempf2025	Abortion care in the emergency department: A national survey of emergency physicians' perspectives	2025	Acad Emerg Med	Kempf AM; Singer MR; Ha ...		PubMed	10/7/25	Y	Institutional/HER	Y	Counseling/delay	Y	Emergency OB (general)	Y	Y		Direct ED clinician mechanisms post-Dobbs	10/7/25
Kennedy2022	Self-testing for pregnancy: a systematic review and meta-analysis	2022	BMJ Open	Kennedy CE; Yeh PT; Gho ...			10/7/25	Unsure		N		N		N	N	No clinical mechanism/outcome	Global review unrelated to Dobbs pipelines	10/7/25
Kerestes2022	Person-centered, high-quality care from a distance: a qualitative study of patient experiences of TelAbortion	2022	Perspect Sex Reprod Health	Kerestes C; Delafield R; Elia ...			10/7/25	Unsure		N		N		N	N	No clinical mechanism/outcome	Teleabortion care model; not emergency OB mechanisms	10/7/25
Khanna2022	Protecting reproductive health information in the post-Roe era: interoperability strategies for healthcare institutions	2022	J Am Med Inform Assoc	Khanna RR; Murray SG; ...		PubMed	10/7/25	Y	EHR/HIE/HIPAA/Claims/Apps	Y	Documentation Changes/Counseling Chill (Use generic codes, counseling on apps)	Y	Miscarriage (Missed AB, Incomplete AB)	Y	Y		High-yield pipeline operations for EHR/HIE governance. Advises institutions to use generic ICD/CPT codes and modify EHR documentation due to interoperability risk.	10/7/25
Killinger2022	Why women choose abortion through telemedicine outside the formal health sector in Germany: a mixed-methods study	2022	BMJ Sex Reprod Health	Killinger, K., Günther, S., Gomperts, R., Atay, H., Endler, M.			10/7/25	N		N		N		N	N	Not U.S.	Germany study	10/7/25
Kirkpatrick2024	Communication and Counseling Preferences of Women Who Chose Abortion During Adolescence: A Qualitative Study	2024	J Pediatr Adolesc Gynecol	Kirkpatrick LA; Bell LA; Ha ...			10/7/25	Unsure		N	Counseling (preferences)	Y		N	N	No relevant data pipeline	Counseling prefs without data-governance or emergency context	10/7/25
Kushnir2024	Preimplantation sex selection via in vitro fertilization: time for a reappraisal	2024	F&S Reports	Kushnir V; Adashi E; Coh ...			10/7/25	Unsure		N		N		N	N	Not emergency OB	IVF ethics; off-scope	10/7/25
Laine2023	Annals for Educators - March 2023	2023	Ann Intern Med (Educators)	Laine C			10/7/25	Unsure		N		N		N	N	Not empirical	Educator column; not a study	10/7/25
LaPaugh2022	Reproductive Health Data as an Evidentiary Tool in State Criminal Prosecutions	2022	Health Law	LaPaugh E		HeinOnline	10/7/25	Y	HIPAA / EHR / law-enforcement subpoena pipeline (discusses state access to reproductive PHI through HIPAA exemptions: “required by law,” “court order,” “law enforcement,” and “civil subpoena”)	Y	Documentation & disclosure chill (providers fear legal liability for releasing or withholding PHI; patients withhold information; uncertainty over disclosure obligations)	Y		N	N	Legal student note reviewing HIPAA exemptions; duplicates content covered by higher-tier analyses	Analyzes HIPAA loopholes enabling state subpoenas for reproductive PHI and the resulting chilling effect on healthcare documentation and data sharing.	10/7/25
Larkin2022	How the Dobbs Decision Shapes Postpartum Permanent Contraception Decision-Making Among Birthing People and Their Clinicians	2022	Journal of Women's Health	Larkin S; Bullington B ...			10/7/25	Y		N	Counseling changes and decision-making shifts but not mechanisms affecting clinical workflow	Y		N	N	No surveillance pipeline and no practice-changing mechanism	Qualitative attitudes on contraception post-Dobbs without pipeline or clinical mechanism.	10/7/25
Leap2023	Life in Emergistan	2023	Emergency Medicine News	Leap E			10/7/25	Y		N		N		N	N	Not empirical	Opinion/news column	10/7/25
Lee2024	HIPAA: Can the Privacy Rule Save the Patient-Physician Relationship in a Post-Dobbs World?	2024	UC Irvine Law Review	Lee LS		HeinOnline	10/7/25	Y	HIPAA (Focus on history/background of Dobbs)	Y	Delay/Disclosure Chill (References doctors facing an ‘impossible choice’ when providing care post-Dobbs)	Y		N	N	Student-authored note. Analysis of HIPAA's role in the provider-patient relationship is covered in greater depth by Francis.	Discusses the legal environment post-Dobbs and the "impossible choice" faced by providers	10/7/25
Lindsey2023	"I can be pro-abortion and pro-birth": Opportunities and challenges for full spectrum care among doulas in Georgia	2023	Front Glob Womens Health	Lindsey A; Narasimhan S; Sa ...		Unknown	10/7/25	Y		N	Counseling (doula)	Y		N	N	No relevant data pipeline	Doula perspectives; lacks EHR/HIE/HIPAA/LE pipeline	10/7/25
Lockett2024	Lived experiences of women with spontaneous abortion at a district hospital, South Africa	2024	S Afr Fam Pract	Lockett M; Mash RJ			10/7/25	N		N		N	Spontaneous abortion (non-U.S.)	N	N	Not U.S.	Non-U.S. qualitative	10/7/25
LongKellough2024	"If I Die, Delete My Browser History": The Fourth Amendment Implications of Reverse Keyword Search Warrants	2024	Capital University Law Review	Long-Kellough A		HeinOnline	10/7/25	Y	Keyword warrants/ geofence warrants; apps	Y	no clinical practice mechanism, no provider-side practice changes	N		N	N	Policy/legal analysis only; no clinical mechanism or OB outcomes	Discusses the potential for abuse of reverse keyword warrants and how Google’s data can help states track abortions post-Roe. Legal commentary; does not involve clinical data, provider behavior, or OB emergency cases.	10/7/25
Madera2025	Digital and Data Security for Abortion Research in a Post‚ÄëDobbs Era: A Primer for Qualitative Researchers	2025	Contraception	Madera M; Bennet A; Bertash K; ...			10/7/25	Y	(research data security)	N		N		N	N	No clinical mechanism/outcome	Research data security primer; not clinical ops	10/7/25
Marino2023	Staying a Jane Doe Post Dobbs and Roe: The Risk Modern Technology Poses with Archaic Abortion Restrictions	2023	Nw. J. Tech. & Intell. Prop.	Marino GE		HeinOnline	10/7/25	Y	ALPR / Facial Recognition / Cell-Site / Internet Search Logs — discusses automatic license-plate readers, facial-recognition software, cell-phone location pinging, and search-engine data collection used in law enforcement post-Dobbs	Y	Legal / surveillance mechanisms affecting practice — explains how law-enforcement access to digital data (ALPR, cell-site, geotagging, search queries) creates chilling effects on patients and providers and impacts care-seeking behavior	Y		N	N	Student-Authored Note/J.D. Candidate. Focuses heavily on the general threat of technology and archaic laws, duplicating themes in Dellinger & Pell.	Post-Dobbs law-review analysis of digital surveillance pipelines (ALPR, FRT, cell-data, search logs) and their use by law enforcement in abortion prosecutions	10/7/25
Martinez2023	Reconsidering the use of urine drug testing in reproductive settings	2023	Am J Obstet Gynecol MFM	Martinez NG; Roberts SC ...		PubMed	10/7/25	Y	Law-enforcement/child-welfare toxicology reporting pathway	Y	Documentation changes, care denial, reporting-driven practice constraints	Y		N	Y		Analyzes how urine toxicology creates a surveillance pipeline and alters reproductive clinical practice, including care denial and legal exposure.	11/23/25
Mastronardi2024	Assessing Pregnant Patients' Postpartum Contraceptive Plans After the SCOTUS Dobbs Decision in a Highly Restrictive State	2024	Obstetrics & Gynecology	Mastronardi, A., Young, M., Oyedeji, O., King, R., Maples, J. & Zite, N.		PubMed	10/7/25	Y	EHR (electronic health record data extraction)	Y	Counseling patterns implied but not described; no mechanism studied	Y		N	N	No mechanism impacting practice and no E-OB clinical outcomes	EHR used only as data source without surveillance or practice-change mechanism.	10/7/25
McKetchnie2025	"I Feel Like there's a Politician in the Room": Provider Perceptions of the Impacts of State Abortion Bans on Physician-Patient Care	2025	Soc Work Public Health	McKetchnie SM; Klein EK; ...			10/7/25	Y	EHR/Documentation/Chart Protection	Y	Counseling/Disclosure Chill	Y	Emergency OB (general)	Y	Y		Direct clinician mechanisms incl. documentation/approvals, documents severe professional consequences and distress	10/7/25
Meriwether2025	Ob/Gyn Trainee Insights Regarding the Effect of the Dobbs Supreme Court Decision	2025	Journal of Women‚Äôs Health	Meriwether KV; Beckham ...		PubMed	10/7/25	Y		N	identifies training limitations, care restrictions, safety concerns, and changes in management of pregnancy and family planning as mechanisms altering how clinicians can provide care.	Y		N	N	Focuses on educational/training perceptions rather than patient-care mechanisms or data pipelines.	Post-Dobbs national trainee survey describing mechanisms—training restrictions and care barriers affecting OBGYN clinical practice.	10/7/25
Mezrich2025	Practicing in the Post-Dobbs Era: New Legal Challenges Facing Radiologists	2025	Acad Radiol	Mezrich JL		PubMed	10/7/25	Y	EHR/PHI (Legal risks/ethical dilemmas for clinicians; focus on reporting lexicon)	Y	Delay/Time-to-Intervention (References medical uncertainty in the shadow of Dobbs when treating obstetric complications)	Y	Ectopic Pregnancy (References first-trimester US, including ectopic pregnancy)	Y	Y		Discusses the legal risks and uncertainty for clinicians, including radiologists, in managing obstetric complications like ectopic pregnancy post-Dobbs	18-Oct
Minnihan2025	Fertility under fire: navigating patient counselling in a post‚ÄëDobbs world	2025	Fertility and Sterility	Minnihan A; Bosch A; ...		PubMed	10/7/25	Y	references patient-physician data exchange and clinical documentation	Y	Counseling - centers on how Dobbs and embryo-personhood laws reshape provider counseling, informed-consent discussions, and patient decision-making in fertility clinics	Y		N	Y		Commentary summarizing post-Dobbs studies showing altered fertility-clinic counseling and patient decision-making regarding embryo use and PGT-A; mechanism = counseling change.	18-Oct
Mosesson2022	Abortion attempts without clinical supervision among transgender, nonbinary and gender‚Äëexpansive people in the United States	2022	BMJ Sex Reprod Health	Mosesson H; Fix L; Gerdts C ...		PubMed	10/7/25	Y		N		N		N	N	No clinical mechanism/outcome	Access/behavior study; not pipeline or emergency OB mechanisms	10/7/25
Moulton2025	A nurse-led model of care to improve access to contraception and abortion in rural general practice: Co-design with consumers and providers	2025	J Adv Nurs	Moulton JE; Arefadib N; Bot ...			10/7/25	N		N		N		N	N	Not U.S.	Likely Australia/UK ‚Äògeneral practice‚Äô context	10/7/25
Munnangi2025	"One's life becomes even more miserable when we hear all those hurtful words". A mixed methods systematic review of disrespect and abuse in abortion care	2025	Front Reprod Health	Munnangi M; Shreedhar P; ...			10/7/25	Unsure		N		N		N	N	No clinical mechanism/outcome	Stigma/harms synthesis; not clinical ops or data pipelines	10/7/25
Nambiar2023	Maternal Morbidity and Fetal Outcomes Among Pregnant Women at 22 Weeks‚Äô Gestation or Less With Complications in the Post‚ÄëDobbs Era	2023	Obstetrics & Gynecology (?), exact journal unclear	Nambiar A; Patel S; ...		PubMed	10/7/25	Y	EHR-based chart abstraction – cases identified from electronic health records and direct patient care	Y	Legal-policy mechanism = forced expectant management and delay in intervention; clinicians required MFM leadership review before acting due to felony risk	Y	PPROM 93%, chorioamnionitis 36%, hemorrhage, ICU admission, hysterectomy	Y	Y		Empirical EHR study showing post-ban legal-policy mechanisms (mandatory expectant management) causing delays, infection, and severe maternal morbidity in Texas hospitals.	18-Oct
Nasrolahi2023	Data surveillance and abortion bans after Dobbs	2023	Berkeley Tech. LJ	Nasrolahi L		HeinOnline	10/7/25	Y	Yes – extensive coverage of apps, geofence warrants, keyword warrants, location data, and social-media data	Y	Indirectly – discusses chilling effects on clinical decision-making and legal information sharing rather than formal approval processes	Y		N	Y		Argues that post-Dobbs abortion enforcement will rely on digital data pipelines (search, GPS, apps) causing chilling effects on legal abortion and healthcare.	18-Oct
Neiman2025	Use of period- or fertility-tracking technologies pre- and post-Dobbs	2025	Contraception	Neiman E; Bornstein M; Turn ...		PubMed	10/7/25	Y	Apps (Period- or fertility-tracking technologies)	Y	Disclosure Chill (Referencing data privacy concerns related to potential prosecution)	Y		N	Y		Study assessing privacy concerns related to period- or fertility-tracking technologies following the Dobbs decision	18-Oct
Nelson2022	Use of period- or fertility-tracking technologies pre- and post-Dobbs	2022	Cancer Cytopathology	Nelson B; Faquin W		PubMed	10/7/25	Y		N		N		N	N	Not empirical	Editorial; background only	10/7/25
Obure2024	"Sometimes you have knowledge but lack the equipment to save a life": barriers to post-abortion care in Liberia and Sierra Leone	2024	Arch Public Health	Obure VA; Juma K; Athero ...			10/7/25	N		N		N		N	N	Not U.S.	Kenya health‚Äësystem barriers	10/7/25
Ong2023	My Body, My Choice, My Data: Information Privacy After Dobbs	2023	USFL Rev.	Ong MK		Google Scholar	10/7/25	Y	Mentions privacy/data pipelines conceptually (HIPAA gaps, data brokers, tech privacy) but not an operational pipeline used in clinical or enforcement setting	Y	None; no provider- or patient-level practice mechanism	Y		N	N	Policy-only; no clinical mechanism/E-OB	Legal analysis of information-privacy rights post-Dobbs; lacks provider-facing or patient-care mechanism	10/7/25
Ouma2025	Telehealth abortion services via Women on Web in Kenya (2013‚Äì2019): descriptive analysis	2025	Sex Reprod Health Matters	Ouma MA; Alaze A; Juma ...			10/7/25	N		N		N		N	N	Not U.S.	Kenya/2013‚Äì2019; outside scope	10/7/25
Oumano2023	Antiabortion laws and reproductive health information in nuclear medicine	2023	Clinical Imaging	Oumano M; Dibble E		PubMed	10/7/25	Y	EMR/HIPAA — discusses how reproductive health information (RHI) recorded in electronic medical records could be subpoenaed or disclosed to law enforcement under HIPAA exceptions	Y	Documentation — recommends reducing collection/documentation of pregnancy information to avoid legal risk; changes in how RHI is recorded and reported under internal policy	Y		N	Y		Post-Dobbs discussion of HIPAA and EMR documentation risks; advises NM clinicians to reduce reproductive data collection to avoid incrimination risk — clear pipeline (EHR/HIPAA) and mechanism (documentation change) present.	18-Oct
Park2023	Reproductive Health Care Data Free or for Sale: Post‚ÄëRoe Surveillance and the ‚ÄúThree Corners‚Äù of Privacy Legislation	2023	Richmond Journal of Law & Technology	Park E		HeinOnline	10/7/25	Y	Geofence warrants, subpoenas, data brokers, period-tracking app data (conceptual discussion only)	N	None empirically. No documentation, delay, counseling change, attestation, or care modification measured.	N		N	N		Privacy law mapping; include if care‚Äëdata custody/disclosure is operationalized	10/7/25
Patronas2025	Bans off our Bodies: Categorizing and Analyzing Reproductive Justice Street Art Across the U.S. (June 2022‚Äì‚Ä¶)	2025		Patronas E	DOI: 10.59236/ne111-263328		10/7/25	Y		N		N		N	N	Not empirical	Art analysis; not clinical or data governance	10/7/25
Paynter2025	The experiences of family planning health professionals providing care to incarcerated patients: a qualitative study	2025	BMC Pregnancy & Childbirth	Paynter M; Heggie C; McLeod ...			10/7/25	N	Docs/Approvals (carceral)	Y	Counseling/Docs (verify)	Y		N	N	Canada	linked documentation/consent issues arise in carceral care	10/7/25
Pearson2024	Effectiveness of the ARCHES intervention among abortion clients	2024	EClinicalMedicine	Pearson E; Paul D; Menzel J ...		PubMed	10/7/25			N		N		N	N	No clinical mechanism/outcome	Intervention effectiveness unrelated to Dobbs/evidence of pipelines	10/7/25
Pinto2023	Geolocation Data and the Fourth Amendment: Geofence Warrants in a Post‚ÄëRoe World	2023	Colo. Tech. L.J.	Pinto M		HeinOnline	10/7/25	Y	Geofence warrants / location data	Y		N		N	N	Policy/legal analysis only; no clinical pipeline–mechanism pathway.	Law review discussing legality of geofence warrants; no clinical practice impact, no mechanisms, and no E-OB cases.	10/7/25
Podolskyi2023	Prelabor rupture of membranes ‚Äî a patient perspective	2023	Eur J Contracept Reprod Health	Podolskyi, V., Gemzell-Danielsson, K., Marions, L., Gomperts, R.			10/7/25			N		N		N	N	Not U.S.	General PROM perspectives; not Dobbs/pipeline specific	10/7/25
Prince2023	Reproductive health surveillance	2023	BCL Rev.	Prince AER		HeinOnline	10/7/25	Y	HIPAA	Y	counseling / disclosure chill	Y		N	Y		Discusses the threat of "uterus surveillance" and provides security and privacy tips for people seeking an abortion	18-Oct
Qaderi2023	Abortion services during the COVID‚Äë19 pandemic: a systematic review	2023	Reprod Health	Qaderi K; Khodavirdilou R; ...			10/7/25			N		N		N	N	No clinical mechanism/outcome	COVID‚Äëera review; not Dobbs or clinical pipeline	10/7/25
Quasebarth2024	Patient experiences using public and private insurance coverage for abortion in Illinois: Implementation successes and challenges	2024	Perspect Sex Reprod Health	Quasebarth M; Boesche M; ...			10/7/25	Y		N		N		N	N	No clinical mechanism/outcome	Insurance coverage experiences; not emergency OB or data pipeline	10/7/25
Raymond2023	Implications for Prenatal Genetic Testing in the United States After the Reversal of Roe v Wade	2023	Obstet Gynecol	Raymond MB; Barbera JP; ...		PubMed	10/7/25	Y		N	counseling constraints due to gestational-age bans	Y		N	N	No pipeline described	Commentary on legal–testing timing; no surveillance/data pipeline	10/7/25
Reamer2023	Ethical Practice in a Post-Roe World: A Guide for Social Workers	2023	Social Work	Reamer FG			10/7/25	Y		N		N		N	N	Not empirical	Professional guidance; not clinical decision-making or pipelines	10/7/25
Reardon2024	Turnaway Study Report Unethically Violated Participants' Privacy and Misleads Public with a Non-Representative Sample	2024	Issues in Law & Medicine	Reardon DC			10/7/25	Y		N		N		N	N	Not empirical	Polemic/critique; no clinical ops	10/7/25
Reingold2022	Legal risks and ethical dilemmas for clinicians in the aftermath of Dobbs	2022	JAMA	Reingold RB; Gostin ...		PubMed	10/7/25	Y	Surveillance, insurer data, app data, social media, law-enforcement access	Y	Delays, refusal of care, clinicians waiting until critical illness, documentation-related fear	Y	Ectopic pregnancy delays, septic miscarriage delays	Y	Y		Includes surveillance pipelines and clear mechanisms delaying E-OB care under Dobbs.	18-Oct
Rivera2023	Hey Siri, Can I Go to Prison for Tracking My Period?: Femtech Apps and Subpoena Powers on Collected Reproductive Health Data	2023	U. Ill. J.L. Tech. & Pol'y	Rivera B		HeinOnline	10/7/25	Y	Apps/Subpoenas	Y		Y		N	N	Policy + legal privacy analysis only; no clinical mechanisms and no E-OB outcomes.	Femtech data + subpoena discussion without clinical impact; pipeline alone insufficient per criteria.	10/7/25
Roper2022	Impact of State Abortion Policies on Family Medicine Practice and Training After Dobbs v Jackson Women‚Äôs Health Organization	2022	Ann Fam Med	Roper KL; Robbins SJ; Day ...		PubMed	10/7/25	Y		N		N		N	N	No pipeline; no clinical mechanism; no E-OB outcomes	FM practice/training; Policy analysis only; population-level effects not linked to practice or data systems	10/7/25
Rothstein2023	The Illusion of Health Privacy in Obstetrics-Gynecology	2023	Clin Obstet Gynecol	Rothstein MA		PubMed	10/7/25	Y	HIPAA, EHR, mobile health data, law-enforcement access	Y	Documentation and disclosure risk pathways; counseling chill	Y	Miscarriage investigations referenced but not clinical management	N	Y		Includes HIPAA/EHR pipelines and mechanisms that alter disclosure and documentation behavior post-Dobbs.	11/23/25
Sabbath2024	US obstetrician-gynecologists' perceived impacts of post‚ÄìDobbs v Jackson state abortion bans	2024	JAMA Network Open	Sabbath EL; McKetchnie ...		PubMed	10/7/25	Y	Institutional/Transfers/Documentation (Practice in states where abortion was illegal; references miscoding risks)	Y	Delay/Time-to-Intervention (Distress at having to delay essential patient care; patient laboring for 12 hours without intervention)	Y	Emergency OB (general)	Y	Y		Qualitative study detailing OB-GYN distress and the inability to provide standard-of-care, essential care due to abortion bans	10/7/25
Saikia2022	Rethinking Explicit Consent and Intimate Data Collection: The Looming Digital Privacy Concern With Roe v. Wade Overturned	2022	LSE Blog (non-journal)	Saikia B; Doshi K			10/7/25	Y	Apps/Privacy	Y		N		N	N	Conference/Non-article	Blog/think piece; not peer-reviewed	10/7/25
Salvatore2024	Women's comfort with mobile applications for menstrual cycle self‚Äëmonitoring following the overturning of Roe v. Wade	2024	mHealth	Salvatore GM; Bercovitz I; ...		PubMed	45937	Y	Apps; Law-enforcement access to reproductive data	Y	Counseling/disclosure chill; refusal to document; switching to nondigital methods	Y		N	Y		Survey showing post-Dobbs fear of app data, data privacy threats, and practice changes.	18-Oct
Sandoval2025	COVID-19 infection history as a risk factor for early pregnancy loss: results from an EHR‚Äëbased cohort	2025	BMC Medicine	Sandoval MN; Klawans MR; ...		PubMed	10/7/25	Y	EHR (no Dobbs)	N		N		N	N	No clinical mechanism/outcome	COVID EHR risk factor study; unrelated to Dobbs/pipelines	10/7/25
Saxena2025	Impact of Dobbs on Evaluation and Treatment of Ectopic Pregnancy: National Survey of Emergency Physicians	2025	West J Emerg Med	Saxena M; Kass D; Choo E		PubMed	10/7/25	Y	Institutional/EHR (Focus on diagnosis/management; ACOG practice bulletins)	Y	Delay/Time-to-Intervention (References evaluating and treating ectopic pregnancy in the post-Dobbs landscape)	Y	Ectopic pregnancy	Y	Y		National survey assessing the impact of Dobbs on the diagnosis and treatment of ectopic pregnancy by Emergency Physicians	10/7/25
Schwartz2023	Second-Trimester Dilation and Evacuation: A Simulation-Based Team Training Curriculum	2023	MedEdPORTAL	Schwartz L; Pelletier A; ...			10/7/25	Y		N		N		N	N	Not emergency OB	Education/simulation; not decision-making or data pipelines	10/7/25
Seymore2023	Social Costs of Dobbs' Pro‚ÄëAdoption Agenda	2023	SSRN Electronic Journal	Seymore M			10/7/25	Y		N		N		N	N	Not empirical	Preprint/essay; no clinical operations	10/7/25
Shachar2022	HIPAA, privacy, and reproductive rights in a post-Roe era	2022	JAMA	Shachar C		PubMed	10/7/25	Y	HIPAA/EHR	Y	mechanism of clinical operational changes is not discussed; this is a legal policy analysis	N	Mentions miscarriage prosecution risk, but not clinical management	N	N	Policy/legal analysis only; no evidence of pipeline impacting clinical practice behavior or E-OB care mechanisms	Discusses HIPAA limits and surveillance risk but provides no mechanism-based or clinical practice impact data	10/7/25
Shachar2023	Informational Privacy After Dobbs	2023	Alabama Law Review	Shachar C; Zubrzycki C		HeinOnline	10/7/25	Y	Apps/EHR/HIPAA (Cites discussions on data privacy involving period trackers, data breaches, and health data)	Y	Disclosure Chill/Deterrence (Constitutional right to informational privacy in the context of protecting abortion-related medical information)	Y		N	Y		Legal piece arguing for the importance of informational privacy in safeguarding abortion-related medical information post-Dobbs	18-Oct
Shalev2023	Politics of reproduction: a view from Israel on the Dobbs decision	2023	Isr J Health Policy Res	Shalev C			10/7/25	N		N		N		N	N	Not U.S.	International perspective	10/7/25
SharifiHeris2023	Impact of social support and mindfulness in perceived risk of COVID‚Äë19 acquisition and pregnancy outcomes	2023	BMC Psychology	Sharifi-Heris, Z., Amiri-Farahani, L., Shahabadi, Z., Sanaei, M.			10/7/25			N		N		N	N	No clinical mechanism/outcome	COVID psychosocial; not Dobbs/pipeline	10/7/25
Shaw2022	Special Report	2022	Emergency Medicine News	Shaw G			10/7/25	Y		N		N		N	N	Not empirical	News column; not a study	10/7/25
SheridanClay2025	Algorithms, allyship, and advice: A qualitative analysis of fertility tracker marketing	2025	Digital Health	Sheridan Clay K; Ziebland S; ...			10/7/25		Apps	Y		N		N	N	No clinical mechanism/outcome	Marketing analysis; not clinical decision-making	10/7/25
Shubkin2024	Transforming Pediatric Education in Response to Dobbs v. Jackson	2024	Academic Pediatrics	Shubkin CD; Sieplinga K; ...			10/7/25	Y		N		N		N	N	Not emergency OB	Education perspective; off scope	10/7/25
Sicula2024	Legal and Risk Management Considerations for Mental Health Providers When Patients Need or Choose an Abortion	2024	The Mental Health Clinician	Sicula M; Rosenberg M; ...		Google Scholar	10/7/25	Y	HIPAA/Disclosure (cross-specialty). Discusses HIPAA Privacy Rule, PHI disclosure, law-enforcement requests for patient records, risk of subpoenas and compelled disclosure	Y	discusses documentation changes to avoid legal exposure, counseling "chill" + avoidance due to fear of aiding/abetting laws, restrictions on provider speech, mandated reporting interactions, liability shaping clinical decision-making	Y	limited discussion of miscarriage care being obstructed	N	Y		HIPAA/EHR pipeline + strong documentation/counseling-chill mechanisms; directly examines post-Dobbs data-exposure risks for patients and clinicians.	18-Oct
Simon2024	The Dobbs Double Bind: Lessons From Substance Use Disorder on the Conflict Between Privacy and Quality Care	2024	Clin Obstet Gynecol	Simon MA; O'Brian CA; ...		PubMed	10/7/25	Y	EHR/HIPAA/privacy policy pipeline	Y	documentation suppression, fear of sharing records, delays in care	Y	miscarriage, ectopic pregnancy, preeclampsia; risk of delayed treatment	Y	Y		Conceptual but clearly describes EHR/HIPAA pipeline risks + practice-level mechanisms causing delayed miscarriage/ectopic care.	18-Oct
Simons2022	ER Goods	2022	Emergency Medicine News	Simons S			10/7/25	Y		N		N		N	N	Not empirical	News/opinion piece	10/7/25
Slavin2023	Women with substance use disorders are highly impacted by the overturning of Roe v. Wade	2023	J Subst Use Addict Treat	Slavin MN; West BS; Levin ...		PubMed	10/7/25	Y		N		N		N	N	No clinical mechanism/outcome	Access/advocacy focus; not pipelines/ED OB	10/7/25
Soltani2025	The new age of periviability	2025	Curr Opin Obstet Gynecol	Soltani A; Waldrop AR; Hen ...		PubMed	10/7/25	Y		Y	Approvals/Delay (verify)	Y	Discusses periviable PPROM, sepsis risk, complications but not in relation to surveillance or data-driven constraints	Y	N	Does not examine data pipelines or practice-changing mechanisms; clinical review only.	Periviability clinical/ethical review; no surveillance, EHR use, or Dobbs-related care delays tied to data pipelines.	10/7/25
Stanley2023	Abortion Privacy in the Digital Age: An Examination of California's Post-Dobbs Informational Privacy Right	2023	Alb. L.J. Sci. & Tech.	Stanley PH		HeinOnline	10/7/25	Y	discusses state informational privacy rather than actual technical pipelines (EHR/HIE/app data, warrants, etc.)	N		N		N	N	Policy-only essay; no data-use pipeline and no clinical mechanisms	California constitutional privacy analysis; no clinical operations, surveillance pipeline, or E-OB relevance	10/7/25
Starosta2024	Medication abortion for adolescents in the United States: Strengthening the role of pediatric primary care providers	2024	Perspect Sex Reprod Health	Starosta A; Harris J; Gariepy ...		PubMed	10/7/25	Y	commentary; doesn't discuss any technical pipelines	N	Discusses values, training, and medical–legal context, but not documentation changes, approvals, attestation steps, delays in care, or disclosure chill	N		N	N	Commentary only; no data-use pipeline and no clinical mechanisms	Policy/education commentary about adolescent abortion access; lacks pipeline + mechanism criteria	10/7/25
Swanson2023	Effect of recent abortion legislation on Twitter user engagement, sentiment, and expressions of trust in clinicians and...	2023	J Med Internet Res	Swanson K; Ravi A; Saleh S ...		PubMed	10/7/25	Y		N		N		N	N	No clinical mechanism/outcome	Social media analysis; not clinical ops or pipelines	10/7/25
Tamire2024	Mixed-methods approach in evaluating safe abortion care services at public health facilities in North Shewa zone, central Ethiopia	2024	Front Health Serv	Tamire A; Birhanu B; Negash ...			10/7/25	N		N		N	Postabortion care (non-U.S.)	N	N	Not U.S.	Non-U.S. mixed-methods	10/7/25
TobinTyler2024	Protecting Sexual and Reproductive Health Privacy Post-Dobbs	2024	JAMA Intern Med	Tobin-Tyler E; Adashi E		PubMed	10/7/25	Y	HIPAA, law-enforcement access to SRHI, apps/femtech, geofencing	Y	policy discussion only; no practice-level mechanism affecting clinician documentation or care delivery	N		N	N		Federal/state privacy policy overview; pipeline present but no mechanisms affecting practice.	10/7/25
Tovino2024	Aborted Confidentiality	2024	B.C. Law Review	Tovino SA		HeinOnline	10/7/25	Y	EHR/HIPAA; subpoenas	Y	Documentation/Disclosure	Y	Sepsis	Y	Y		Law review; Legal analysis of HIPAA and PHI disclosure risks, citing a case where delayed care for a complication led to septic shock/death	18-Oct
VandeVusse2025	Effects of the Dobbs Decision on Publicly Supported Sexual and Reproductive Health Clinics: Results from a National Study	2025	J Womens Health (Larchmt)	VandeVusse A; Mueller J; Kirk ...		PubMed	10/7/25	Y	N	Y	counseling / disclosure chill ( lower proportions of sites providing pregnancy options counseling and miscarriage management, large proportions of sites chose not to answer pregnancy-related questions)	Y		N	N		no pipeline	10/21/25
Walker2022	Interoperability in a Post-Roe Era: Sustaining Progress While Protecting Reproductive Health Information	2022	JAMA	Walker DM; Hoffman S; Adlin ...		PubMed	10/7/25	Y	EHR/HIE/HIPAA	Y	Disclosure chill / prosecution (HIE networks could facilitate investigation and prosecution of individuals)	Y		N	Y		Discusses the risk that HIE networks and comprehensive records could facilitate investigation and prosecution of individuals who obtained abortion services	10/7/25
Whalen2024	Assessing pharmacist and clinician perspectives on pharmacist-prescribed hormonal contraceptives	2024	J Am Pharm Assoc	Whalen A; Bratberg J; Cohen ...		PubMed	10/7/25			N		N		N	N	No clinical mechanism/outcome	Pharmacist-prescribed contraception; not Dobbs or emergency OB	10/7/25
Willis2024	Evaluating participant engagement in a preconception cohort study in relation to the Dobbs decision	2024	Paediatr Perinat Epidemiol	Willis MD; Hoffman MN; ...		PubMed	10/7/25	Y		N		N		N	N	No clinical mechanism/outcome	Research participation effects; not clinical decision-making	10/7/25
Woldetsadik2024	Exploring barriers to using modern contraceptives and accessing safe abortion care in women who terminated unintended pregnancies	2024	BMC Womens Health	Woldetsadik MA; Yoseph Y; ...			10/7/25	N		N		N	Non-U.S. abortion care	N	N	Not U.S.	Non-U.S. context	10/7/25
Wolf2023	What if We Were Me? A Qualitative Exploratory Study of Emergency Nurses' Clinical Decision Making Related to Obstetric Emergencies	2023	J Emerg Nurs	Wolf L; Noblewolf HS; Callihan ...		PubMed	10/7/25	Y	Institutional/Legal governance pipeline: medicolegal landscape, fear of legal repercussions, uncertainty about reportability	Y	Delay in care due to transfer battles between ED and OB, confusion about reportability (disclosure chill), no guidance, provider fear	Y	miscarriage, previable pregnancy emergencies, suspected infection/sepsis, hemorrhage risk, PPROM-like scenarios	Y	Y		ED nurses describe delays, uncertainty, and clinical chilling effects in managing previable pregnancy emergencies under abortion-ban laws.	18-Oct
Zadushlivy2025	Exploration of Reproductive Health Apps' Data Privacy Policies and the Risks Posed to Users: Qualitative Content Analysis	2025	J Med Internet Res	Zadushlivy N; Biviji R; William ...		PubMed	10/7/25	Y	App data collection, third-party sharing, IP tracking, SDK-based data transfers clearly described	Y	No documentation changes, counseling changes, approval requirements, workflow delays, or clinical decision impacts described. Only consumer-facing data-privacy analysis.	N		N	N	No mechanism affecting clinical practice; no emergency OB content.	App privacy/security evaluation only; no provider practice implications.	10/7/25
